# Predicting Fecundity of Fathead Minnows (*Pimephales promelas*) Exposed to Endocrine-Disrupting Chemicals Using a MATLAB®-Based Model of Oocyte Growth Dynamics

**DOI:** 10.1371/journal.pone.0146594

**Published:** 2016-01-12

**Authors:** Karen H. Watanabe, Michael Mayo, Kathleen M. Jensen, Daniel L. Villeneuve, Gerald T. Ankley, Edward J. Perkins

**Affiliations:** 1 Division of Environmental and Biomolecular Systems, Institute of Environmental Health, and School of Public Health, Oregon Health & Science University, Portland, Oregon, United States of America; 2 Environmental Laboratory, U.S. Army Engineer Research and Development Center, Vicksburg, Mississippi, United States of America; 3 Mid-Continent Ecology Division, U.S. Environmental Protection Agency, Duluth, Minnesota, United States of America; CNRS, FRANCE

## Abstract

Fish spawning is often used as an integrated measure of reproductive toxicity, and an indicator of aquatic ecosystem health in the context of forecasting potential population-level effects considered important for ecological risk assessment. Consequently, there is a need for flexible, widely-applicable, biologically-based models that can predict changes in fecundity in response to chemical exposures, based on readily measured biochemical endpoints, such as plasma vitellogenin (VTG) concentrations, as input parameters. Herein we describe a MATLAB^®^ version of an oocyte growth dynamics model for fathead minnows (*Pimephales promelas*) with a graphical user interface based upon a previously published model developed with MCSim software and evaluated with data from fathead minnows exposed to an androgenic chemical, 17β-trenbolone. We extended the evaluation of our new model to include six chemicals that inhibit enzymes involved in steroid biosynthesis: fadrozole, ketoconazole, propiconazole, prochloraz, fenarimol, and trilostane. In addition, for unexposed fathead minnows from group spawning design studies, and those exposed to the six chemicals, we evaluated whether the model is capable of predicting the average number of eggs per spawn and the average number of spawns per female, which was not evaluated previously. The new model is significantly improved in terms of ease of use, platform independence, and utility for providing output in a format that can be used as input into a population dynamics model. Model-predicted minimum and maximum cumulative fecundity over time encompassed the observed data for fadrozole and most propiconazole, prochloraz, fenarimol and trilostane treatments, but did not consistently replicate results from ketoconazole treatments. For average fecundity (eggs•female^-1^•day^-1^), eggs per spawn, and the number of spawns per female, the range of model-predicted values generally encompassed the experimentally observed values. Overall, we found that the model predicts reproduction metrics robustly and its predictions capture the variability in the experimentally observed data.

## Introduction

Prediction of contaminant impacts on fish populations is an important component of ecological risk assessment. Due to the difficulties associated with directly measuring population-level impacts in the ambient environment, biologically based models are commonly used to estimate potential population-level effects based upon other types of predictive endpoints which can be plausibly and robustly linked to demographic outcomes. One approach has been to use laboratory-based measurements of cumulative fecundity as an input parameter for predicting potential population-level impacts [1]. However, the studies required to generate such data, e.g., from 21-day fish short-term reproduction studies [2], remain relatively costly and time-consuming. Consequently, there is interest in deriving useful predictions of potential population-level impact(s) from other types of measurements that can be made more rapidly and/or cost effectively. These include *in vitro* assays used in chemical toxicity testing [3,4] or organism-specific cell/tissue cultures that could be used to determine changes in key endpoints that are known to elicit adverse effects when perturbed sufficiently. Results from these assays require extrapolation to determine effects upon an organism and ultimately a population, which can be accomplished with mathematical/computational models.

Plasma vitellogenin (VTG) concentration is an example of a biochemical measure that has been proposed as an input parameter for predictive modeling of potential reproductive outcomes in fish. Due to its role as an egg yolk precursor, it can be related to the energy status of developing embryos. Depending on species, VTG can account for up to 90% of the mass or volume of fish oocytes and the uptake of VTG is a central process in fish oocyte maturation [5]. Furthermore, VTG synthesis is known to be impacted by a variety of environmentally-relevant contaminants that affect endocrine function in fish [6–14]. Given this central role in fish reproduction, impaired VTG production in females has been identified as a key event in a variety of adverse outcome pathways (AOPs, https://aopkb.org/) that lead to fish reproductive failure [15]. The ability to predict probable reproductive outcomes in fish (e.g., cumulative fecundity and other reproduction metrics like spawns per female per unit time, eggs per spawning event, etc.) based on plasma VTG concentrations could potentially facilitate the use of shorter-term, more cost effective testing approaches for predicting reproductive outcomes.

To support this aim, Li et al. [16] published an oocyte growth dynamics model for fathead minnows that uses a constant plasma VTG concentration as input. The model developed by Li et al., which has a probabilistic component that randomly samples empirical distributions of unexposed (control) fathead minnow clutch sizes and spawning intervals to determine the number of oogonia recruited into growth and development and the recruitment interval between batches of oogonia, was developed and evaluated using MCSim software [17]. In this biologically-based model, oocyte growth and development is driven by absorption of VTG from plasma. Thus, plasma VTG concentration is an important model input variable. In addition to utilizing information from control animals, Li et al. [16] evaluated performance of the model using data from fish exposed to 17β-trenbolone [18], a steroidal androgen that depresses VTG synthesis in fish.

While MCSim is a powerful Monte Carlo simulation tool, it lacks some of the benefits provided by MATLAB^®^, a more widely used software suite with an integrated capability to develop a graphical user interface (GUI). Thus, to improve user-friendliness and broaden the user base, we encoded the model of Li et al. [16] in MATLAB^®^, developed a GUI, and compiled it as executable programs for Windows XP, Mac or Unix/Linux operating systems (MATLAB^®^ source code available for download at https://github.com/KarenWatanabe/Oocyte_Growth_Dynamics.git; executable code is available upon request from KHW due to file size limitations on GitHub). As such, a MATLAB^®^ license is not required to use the MATLAB^®^ version of the oocyte growth dynamics model (herein labeled OGDM). It is expected that these refinements will enable greater use of this model for predictive ecological assessments.

Nonetheless, before the model can be adopted and accepted for broader use to support environmental decision-making, it is necessary to establish confidence that its predictions are reasonable and provide a level of certainty similar to or better than current methods. To achieve this aim, we evaluated this MATLAB^®^-based OGDM with the data sets used by Li et al. [16] to ensure consistent cross-platform results with the MCSim implementation. Additionally, we assessed the generality of its predictions for endocrine active chemicals with different modes of action. OGDM predictions were compared with several reproduction metrics (e.g., average fecundity, eggs per spawn and spawns per female) obtained from short-term (21 day) reproduction assays with fathead minnows. This standardized assay is being used extensively both in the US and elsewhere for legislatively mandated screening and testing of endocrine-disrupting chemicals [19,20]. Using the 21-day design, fathead minnows were exposed not only to 17β-trenbolone, but to other endocrine active chemicals (for a review see [19]). Of the seven chemicals listed as steroid synthesis inhibitors in Ankley et al. [19], we selected fadrozole, ketoconazole, propiconazole, prochloraz, fenarimol, and trilostane because they that depress VTG production to varying degrees and impact fecundity [6,8,12,13,21].

In the 21-day reproduction study, two types of spawning designs can be used: (i) group spawning with four females and two males per replicate, and (ii) paired spawning with one female and one male per replicate. Li et al. [16] found that their oocyte growth dynamics model based upon clutch size distributions and spawning intervals from control fathead minnows in paired spawning design studies was able to predict average fecundity of control fathead minnows from group spawning design studies. However, the average number of eggs per spawn and average number of spawns per female were not evaluated because the number of eggs spawned by an individual female cannot be determined explicitly in a group spawning design. Assuming plasma VTG concentrations measured at the end of a 21-day study were constant throughout the modeling time period, the OGDM was first used to predict results for fathead minnows exposed to 17β-trenbolone in a group spawning design. Second, OGDM model predictions were evaluated using reproduction metrics for control fathead minnows from 21-day reproduction studies that used the group spawning design [7,8,12,22,23]. Finally, the OGDM was used to predict reproduction metrics of fathead minnows exposed to steroid synthesis inhibitors in group and paired spawning experiments. The goal was to evaluate how well the OGDM predicted empirical outcomes using plasma VTG concentrations, which can feasibly be measured in shorter term tests, as the primary input parameter.

## Materials and Methods

### Development of Oocyte Growth Dynamics Model in MATLAB®

We briefly summarize the mathematical formulation for the oocyte growth dynamics model published by Li et al. [16] that forms the basis for this study. Batches of oocytes grow by absorbing VTG, water and other essential nutrients until they reach a threshold volume required for spawning. The threshold volume (= 0.52 μL) was calculated from the diameter of mature oocytes reported in Leino et al. [24], and all oocytes within a given batch are assumed to grow at the same rate. Following the formulation of Li et al., the equations for the change in oocyte mass and volume are shown in Eqs 1 and 2, respectively.

$$\frac{{dM}_{\text{VTG}}}{dt}=\rho_{\text{VTG\_Ooc}}C_{\text{VTG}}V_{\text{Ooc}}$$ (1)

$$V_{\text{Ooc}}(t)=0.156R_{\text{VTG}}M_{\text{VTG}}(t)+V_{\text{Oog}},$$ (2)

where, *M*_VTG_ (nmol) is the mass of VTG absorbed into an oocyte within a batch, *ρ*_VTG_Ooc_ (h^-1^) is the VTG absorption rate constant, *C*_VTG_ (nmol•μL^-1^) is the plasma VTG concentration, and *V*_Ooc_ (μL) is the oocyte volume. In Eq 2, 0.156 (mg•nmol^-1^) is the molecular mass of VTG, *V*_Oog_ is the oogonium volume (i.e., initial volume of an oocyte), and because oocytes grow by absorbing water and other nutrients in addition to VTG, *R*_VTG_ (μL•mg^-1^) is a ratio of oocyte volume per mass of VTG absorbed. The initial conditions are *M*_VTG_(0) = 0 and *V*_Ooc_(0) = *V*_Oog_, which we assumed to be 3 months of age when oogonia recruitment begins in a sexually-maturing fathead minnow reared at a temperature and photoperiod that is optimal for spawning. To solve the model equations numerically, we used the MATLAB^®^ numerical integration function ODE45 with a relative error tolerance of 1e-3 and absolute error tolerance of 1e-10.

Each simulation begins after an 81-day “start-up” period. This corresponds to 60 days from the start of oogonia recruitment at 3 months to 5-month old fathead minnows typically used in the reproduction experiments, plus a 21-day acclimation/observation (pre-exposure) period prior to the start of a 21-day chemical exposure following the standardized protocol described by Ankley et al. [23]. In the OGDM output files, times reported as negative values correspond to the 81-day start-up period.

The number of oogonia in a batch that are recruited into the growth and development phase was determined by randomly sampling a truncated lognormal distribution that was fit to clutch size data from 391 control fathead minnows in paired spawning design studies (μ = 4.11, σ = 0.96, lower bound = 1, upper bound = 317). Similarly, the time interval between batches recruited into growth and development was determined by randomly sampling a truncated lognormal distribution (μ = 1.21, σ = 0.57, lower bound = 1, upper bound = 25) fit to spawning interval data from 331 control fathead minnows in paired spawning design studies. See Li et al. [16] for a more detailed explanation of these data.

MATLAB^®^ was used to create a GUI for the oocyte growth dynamics model (see S1 Appendix). Up to 1000 fish may be simulated concurrently, and for each fish, plasma VTG concentration (*C*_VTG_, nmol•μL^-1^) is a required input; values may be entered manually or uploaded from a text file through the GUI. The number of simulation days must also be specified by the user in order to run the model.

Three text output files are written by the OGDM to enable calculation and plotting of reproduction metrics (see S1 Appendix). For each fish simulated, a summary of results is reported in text files named OGDM_ResultsSummary_*.csv (* corresponds to the simulation number), that contains the input plasma VTG concentration, oocyte batch sizes and spawning intervals, including batches that are recruited into growth and development, but not spawned. The second output file is OGDM_ClutchSizeSummary.txt, which reports a matrix of clutch sizes for each fish simulated from 81 days pre-exposure to the end of the user-specified simulation period (= 21 days for a 21-day reproduction study). Lastly, OGDM_SpawningSummary.txt reports the total number of spawns and total eggs spawned for (i) the entire simulation period (81-days pre-exposure + the user-specified number of simulation days); and (ii) for just the user-specified number of simulation days. Reproduction metrics can be calculated in multiple ways from the different output files, but each output file presents results in a unique way that is useful for some calculations compared to others. For example, for one fish, the average number of eggs per spawn (total eggs spawned/total number of spawns) can be readily calculated from results within OGDM_SpawningSummary.txt. It can also be calculated from results within OGDM_ClutchSizeSummary.txt by manually summing the eggs spawned in each clutch, then counting the number of clutches and calculating the average number of eggs per spawn. A user with a MATLAB^®^ license may choose to process results within MATLAB^®^, or to import the text files into a spreadsheet or plotting package of their choice. Though only a selection of model predictions are used in the present study, the model can be used to make predictions of a variety of other endpoints, e.g., oocyte mass and volume versus time (in output file OGDM_OocMassVol.csv), growth time for oocyte maturation, and spawning intervals. Supporting information, S1 Appendix, provides descriptions and examples of the model output files.

### Model Evaluation

The OGDM was evaluated in three phases by comparing OGDM predictions with: (i) predictions from the MCSim version [16] for 17β-trenbolone-exposed fathead minnows; (ii) reproduction metrics for control fish from group spawning design studies; and (iii) predictions based on 21-day reproduction studies with four steroid biosynthesis inhibitors that inhibit cytochrome P450 aromatase (CYP19) to varying degrees, and two that affect different enzymes in the steroid biosynthesis pathway. In these evaluations, model predictions were used to calculate a variety of reproduction metrics such as average fecundity, eggs per spawn, spawns per female, and the cumulative number of eggs spawned over time (cumulative fecundity), all of which are endpoints in the standardized 21-day assay used for endocrine disruptor screening and testing [23,25].

Model predictions were post-processed in MATLAB^®^, and scripts (m-files) were created to calculate and plot results for each 21-day reproduction study (m-files are available upon request). Total eggs spawned (*n*_egg___total_) can be obtained directly from the output file OGDM_SpawningSummary.txt. Equations that were used to calculate the reproduction metrics are as follows:

$$\text{Average fecundity}=\left(n_{\text{egg\_total}}/n_{\text{female}}\right)/n_{\text{days}},$$ (3)

where, *n*_female_ is the number of female fathead minnows that produced *n*_egg_total_, and *n*_days_ is the number of days over which *n*_egg_total_ was measured;

$$\text{Eggs per spawn}=n_{\text{egg\_total}}/n_{\text{spawns}},$$ (4)

where, *n*_spawns_ is the total number of spawns that produced *n*_egg_total_; and finally the number of spawns per female is calculated as

$$\text{Spawns per female}=n_{\text{spawns}}/n_{\text{female}}.$$ (5)

For fish that did not spawn during the experimental period, eggs per spawn is mathematically undefined. In our figures, we plotted these undefined values as zeros to indicate that no spawning occurred for at least one fish in that treatment group and to visually anchor low OGDM-predicted values of eggs per spawn. Cumulative fecundity versus time was calculated from results in OGDM_ClutchSizeSummary.txt. The cumulative sum function in MATLAB^®^ was used to calculate cumulative fecundity over the period of interest. For a group spawning design study at one treatment level, the cumulative fecundity on *n*_days_ is the sum of all eggs collected from all groups, *j*, up to that day.

$${\text{Cumulative fecundity}}_{n_{days}}=\sum_{d=1}^{n_{\text{days}}}{\left(\sum_{j=1}^{n_{\text{groups}}}n_{\text{eggs},j}\right)}_{d},$$ (6)

where, *n*_eggs, j_ is the number of eggs collected from a group (typically containing four female fathead minnows), and *n*_groups_ is the number of groups per treatment (typically three or four).

#### Comparison of results from OGDM with MCSim oocyte growth dynamics model

Model predictions were compared to data for fathead minnows exposed to 17β-trenbolone, an anabolic agent used in live stock production that has been identified as an endocrine active environmental contaminant ([26]; trenbolone, CAS number 10161-33-8) in a 21-day reproduction study [7]. We performed 50 simulations of each fish in the experiment (control and five treatments, *n* = 71 fish total) using the MCSim oocyte growth dynamics model [16]. We used a nonparametric, two-sample Kolmogorov-Smirnoff test to test the hypothesis that the OGDM-predicted distribution of total eggs spawned by each fish in the 21-day exposure period was the same as the distribution predicted using the MCSim version. In MATLAB^®^, we implemented the kstest2 function to perform the analysis at a significance level, α, of 0.05.

#### Evaluation with data from control fathead minnows in group spawning design studies

OGDM predictions were evaluated with reproduction metrics derived from 74 control fathead minnows from six 21-day reproduction studies with endocrine active compounds: 17β-trenbolone [7]; fadrozole [8]; flutamide [22]; methoxychlor and methyltestosterone [23]; and ketoconazole [12]. These studies used a group spawning design and thus, reproduction metrics for the 19 groups of fish (17 groups of four; two groups of three females) from these experiments were compared with OGDM predictions. Previously, Li et al. [16] evaluated average fecundity model predictions, but did not evaluate predictions of the average number of eggs per spawn or the average number of spawns per female because the oocyte growth dynamics model is based upon clutch sizes and spawning intervals from *paired* spawning design studies. Average fecundity only requires knowledge of the total number of eggs spawned over the study period, and Li et al. showed that their model adequately predicts average fecundity from group spawning design studies. In addition to average fecundity, we evaluated how well the OGDM predicts the average number of eggs per spawn, and the average number of spawns per female from group spawning design studies. To reconcile OGDM predictions based upon paired spawning design studies with experimental data from group spawning design studies, we hypothesized that if multiple fish in a group spawn on the same day, then these eggs can be viewed as originating from one spawn with an egg count equal to the sum of eggs from each fish on that day. Thus, we simulated each fish in a group separately based on its measured plasma VTG concentration, grouped fish according to the experimental design, and “binned” spawning events by study day. Binned spawn counts were then used to calculate the average number of eggs per spawn and the average number of spawns per female. S1 Table contains the measurements of plasma VTG concentrations, experimentally derived reproduction metrics, and their sources. The two-sample Kolmogorov-Smirnoff test was used to evaluate whether the distributions were different at α = 0.05.

#### Evaluation with data from fathead minnows exposed to steroid biosynthesis inhibitors

Fadrozole hydrochloride (CAS number 102676-31-3) is a highly specific, reversible, competitive nonsteroidal inhibitor of CYP19, which was used in Japan as a treatment for estrogen-dependent breast cancers [27]. Fathead minnows were exposed to four different fadrozole treatments (0 [control], 2, 10 or 50 μg•L^-1^) for 21 days in a group spawning design [8]. Each treatment level included three replicate tanks containing four female and two male adult fathead minnows. Significant decreases in plasma VTG and fecundity were reported for all exposure concentrations [8], though hatching success was not significantly affected.

Ketoconazole (CAS number 65277-42-1) is a pharmaceutical fungicide used to treat skin and mucous membrane infections. In fungi and yeast, ketoconazole inhibits 14α-lanosterol demethylase (CYP51) to prevent the formation of ergosterol. In mammals, ketoconazole has been shown to inhibit a variety of steroidogenic CYP enzymes including steroid 17α-hydroxylase and/or 17,20-lyase (CYP17), steroid 11β-hydroxylase (CYP11B), and CYP19 [28]. In addition, ketoconazole reversibly inhibits xenobiotic metabolizing enzymes, aryl hydrocarbon hydroxylase (CYP1A1) and cytochrome P450, Family 3, Subfamily A (CYP3A) [12]. Fathead minnows were exposed to five different ketoconazole treatments (0, 6, 25, 100, and 400 μg •L^-1^) for 21 days in a group spawning design with four replicate tanks per treatment. At 25 and 400 μg•L^-1^, the mean number of spawns per female and the average fecundity were significantly lower compared to the controls. Fertility and hatching success were unaffected by the ketoconazole exposures.

Propiconazole (CAS number 60207-90-1) is a fungicide used in agricultural applications. Skolness et al. [13] performed a 21-day reproduction study with five treatments (i.e., 0, 5, 50, 500 and 1000 μg •L^-1^) in a paired spawning design (12 pairs per treatment level). They reported that egg production was reduced at the 500 and 1000 μg•L^-1^ treatment levels, but fertility and hatching success were not adversely affected by the propiconazole exposures.

Prochloraz (CAS number 67747-09-5) is an imidazole fungicide like ketoconazole, which inhibits CYP17 and CYP19 in mammals and fish [6,29] and is also an androgen receptor antagonist. Ankley et al. [6] performed a 21-day study using a paired spawning design with a eight pairs of control fathead minnows and three prochloraz treatment groups (30, 100, and 300 μg •L^-1^). Concentration dependent decreases in fecundity were observed for both chemicals, but hatching success and fertility were not affected.

Fenarimol (CAS number 60168-88-9) is also a fungicide used agriculturally to treat moldy seeds [30]. Like prochloraz, *in vitro* assays indicate that fenarimol inhibits CYP19 and is an antagonist of the androgen receptor in mammals and fish [6]. In parallel with the prochloraz study, Ankley et al. [6] exposed four pairs of fathead minnows to two fenarimol treatments (100 and 1000 μg •L^-1^). The observed collective effects of fenarimol upon different reproductive endpoints was unlike collective effects observed in other 21-day reproduction studies with CYP19 inhibitors or anti-androgens. However, to evaluate whether the OGDM would be able to predict egg production for endocrine active chemicals with slightly different modes of action, we performed simulations of the 21-day reproduction data for fenarimol and trilostane.

Trilostane (CAS number 13647-35-3), is a competitive inhibitor of 3β-hydroxysteroid dehydrogenase (3β-HSD) that is used to treat Cushing’s syndrome, a condition involving elevated cortisol levels [31]. In steroid biosynthesis, 3β-HSD catalyzes the conversion of pregnenolone to progesterone, or more generally Δ^5^–3β-hydroxysteroids to Δ^4^-3-ketosteroids [21]. Villeneuve et al. [21] conducted a 21-day reproduction study using the paired spawning design with 14 controls and 10 pairs of fathead minnows per treatment group (nominal exposure concentrations of 60, 300 and 1500 μg •L^-1^). Plasma VTG concentrations were affected in a concentration-dependent manner and fecundity at the highest trilostane concentration was significantly reduced.

Reproduction metrics from the six studies described above were used to evaluate OGDM predictions. For each fish in the experiments, we performed 50 OGDM simulations using a measured plasma VTG concentration. If plasma VTG concentration was not measured for a fish, we excluded the fish from our simulations, but if spawning data were available for the same fish the data were included in the calculation of experimental reproduction metrics. For group spawning design studies, simulated fish were assigned according to their experimental grouping and reproduction metrics were calculated with Eqs 3 to 6. For a single treatment with three replicate groups of four fish per group, 600 OGDM simulations were performed, and reproduction metrics were calculated from the OGDM predictions resulting in 50 values of each metric per group (150 values for a treatment). Due to the small number of experimentally derived reproduction metrics from the 21-day reproduction studies, we evaluated model predictions by plotting the measured endpoints over boxplots of OGDM predictions.

The OGDM source code and plasma VTG concentration input files corresponding to all the simulations performed herein are available for download at https://github.com/KarenWatanabe/Oocyte_Growth_Dynamics.git. Executable versions of the OGDM are available upon request from KHW.

## Results and Discussion

### Comparison with MCSim oocyte growth dynamics model

**Fig 1:**
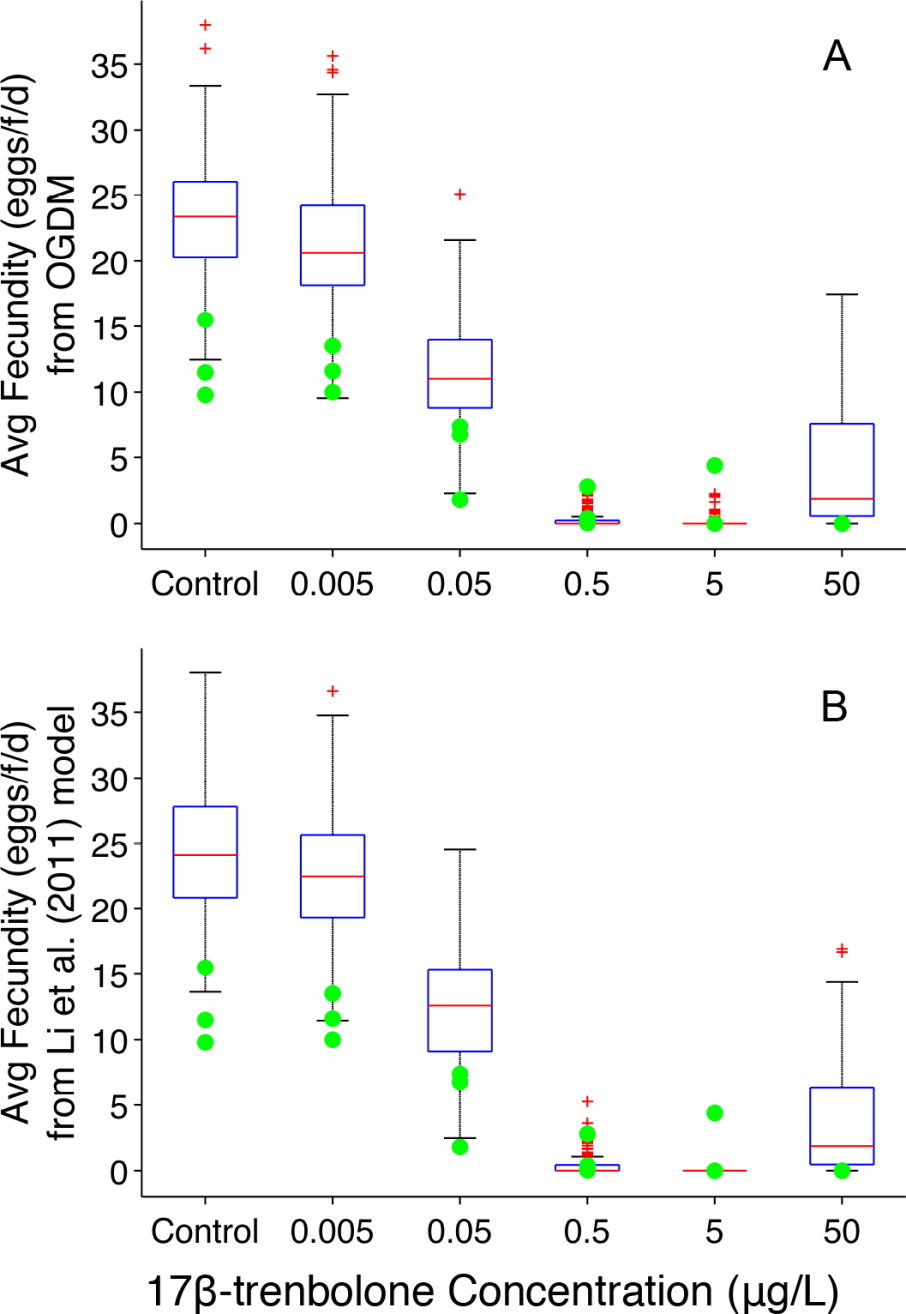
Predicted Average Fecundity for Fathead Minnows Exposed to 17β-trenbolone (group spawning design). (A) OGDM model predictions. (B) MCSim oocyte growth dynamics model predictions. Filled circles represent experimentally observed values [18]. Boxplots represent 150 model-simulated values (three groups of fish per treatment simulated 50 times per group). In the boxplots, the red line represents the median, lower and upper edges of the box are the 25^th^ and 75^th^ percentiles, respectively, lower and upper whiskers denote the most extreme values that are not outliers (~2.7σ or 99.3 percentile for normally distributed values), and the red + symbol represents outliers.

The MCSim oocyte growth dynamics model and the OGDM yield similar predictions of total eggs spawned and, ultimately, of average fecundity with slight differences attributable to the probabilistic nature of the models (Fig 1). Thus, we are confident that translation of the MCSim oocyte growth dynamics model into MATLAB^®^ is error-free. There are, however, differences in functionality such as (i) a user’s ability to modify default parameter values; (ii) user-friendliness; and (iii) compatibility with different operating systems. Despite these differences, predictions from both versions of the model can be expected to provide comparable results given identical input parameter values.

With respect to modifying default parameter values, the MCSim oocyte growth dynamics model has greater flexibility through the user-defined model input file. Any model parameter can be redefined at run-time through the model input file. In contrast, the GUI of the OGDM gives users the ability to input only the simulation length and plasma VTG concentration(s) during the simulation period, but not for the 81-day pre-exposure period. We encountered this difference in our first set of model comparisons for 17β-trenbolone exposed fish. While both models have a default plasma VTG concentration for unexposed fathead minnows in the pre-exposure period based upon experimental measurements in control fish (= 0.1 nmol•μL^-1^), Li et al. [16] redefined these values to better reflect the data set being simulated. For each control fish, the pre-exposure plasma VTG concentration (CVTG_gon_base in the model input file) was set to the measured plasma VTG concentration at the end of the study, and for all 17β-trenbolone exposed fish, CVTG_gon_base was set to the average plasma VTG concentration of the controls (= 0.1725 nmol•μL^-1^). This resulted in statistically significant differences when results were compared to the OGDM. Thus, for our model comparison of 17β-trenbolone exposed fish, the MCSim version was run with CVTG_gon_base = 0.1 nmol•μL^-1^ for all simulated fish.

In terms of user-friendliness, the OGDM offers a GUI that is not available for the MCSim version. The GUI provides a simple interface that guides users through the process of running a simulation for up to 1000 fish at one time. In contrast, the MCSim version does not have an upper limit on the number of fish that can be simulated at one time, but all model output (i.e., values for each fish simulated and each user-defined endpoint) is written to the same output file. As described earlier, the OGDM produces three different types of output files to facilitate the calculation of various reproduction metrics (see S1 Appendix). Output files from both models can be imported into other software packages for further analysis and data visualization.

Finally, to run the MCSim version, a user needs a Linux/Unix operating system. The OGDM is available to run on Windows XP, Mac OS, and Linux operating systems, and users do not need a MATLAB^®^ license to run the packaged executable OGDM (available upon request). Analysis of model predictions in MATLAB^®^ would require a license, but the same would be true for output from the MCSim version.

#### Fathead minnows exposed to 17β-trenbolone

We found that the majority of the total eggs spawned distributions (68 of 71) predicted by the MCSim oocyte growth dynamics model and the OGDM were not significantly different. However, for three fish the distributions of total eggs spawned were found to be significantly different: two out of 12 in the 0.005 μg trenbolone•L^-1^ treatment (*p*-values = 0.002 and 0.03); and one out of 12 in the 0.05 μg trenbolone•L^-1^ treatment (*p*-value = 0.03). This is reasonable given the probabilistic nature of the oocyte growth dynamics model.

In Fig 1, with the exception of the 0.5 and 5 μg trenbolone•L^-1^ treatments, the lower values of both sets of model predictions overlap the experimentally observed average fecundity values, and median model predictions from both models are greater than the experimentally observed values (Table 1). For the 0.5 and 5 μg•L^-1^ treatments, most model predictions were equal to zero (*n* = 108 and 131, respectively); nonzero values ranged from 0.095 to 2.9, and 0.13 to 2.3, respectively. These results confirm that the OGDM produces comparable results to the MCSim version of the model, and validates that the mathematical and computational implementation of the model is error-free.

**Table 1:** Average fecundity (eggs•female^-1^•day^-1^) of 17β-trenbolone exposed fathead minnows: experimentally observed values, and median predictions from the OGDM and MCSim oocyte growth dynamics models.

		17β-trenbolone Concentration (μg•L^-1^)					
	Replicate	0	0.005	0.05	0.5	5.0	50
**Experimentally observed**	1	12	10	1.8	0.04	0	0
	2	16	12	7.4	0.40	4.4	0
	3	10	14	6.8	2.8	0	0
**MCSim**	median	24	23	13	0	0	1.9
**OGDM**	median	23	21	11	0	0	1.9

### Prediction of spawning for control fathead minnows from group spawning design studies

**Fig 2:**
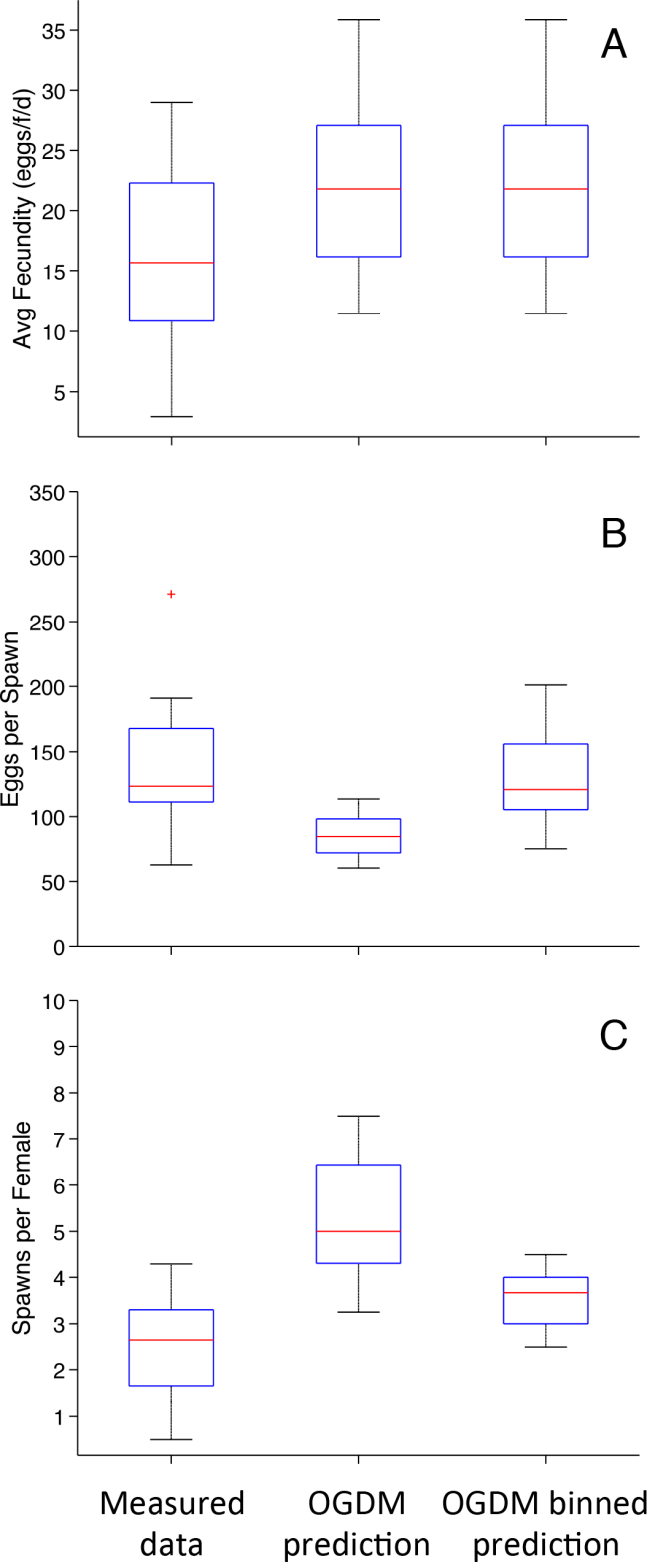
Comparison of Reproduction Metrics for Control Fathead Minnows in a Group Spawning Design. Experimentally observed values, OGDM predictions (spawns and eggs counted separately for each fish in a group), OGDM binned predictions (multiple fish in a group spawning on the same day are summed and counted as one spawn with an egg count equal to the sum of eggs that day). (A) Average fecundity (*n* = 19). (B) Average number of eggs per spawn (*n* = 16). (C) Average number of spawns per female (*n* = 16). In the boxplots, the red line represents the median, lower and upper edges of the box are the 25^th^ and 75^th^ percentiles, respectively, lower and upper whiskers denote the most extreme values that are not outliers (~2.7σ or 99.3 percentile for normally distributed values), and the red + symbol represents outliers.

OGDM-predicted average fecundity was not significantly different from experimentally observed values with or without binning the daily eggs spawned by multiple fish in a group and counting it as one spawn (Fig 2A; *p*-values = 0.12 for both methods of calculation). For the average number of eggs per spawn (Fig 2B, OGDM Prediction) and spawns per female (Fig 2C, OGDM Prediction), the distributions were found to be significantly different without binning (*p*-values = 1.3 × 10^−4^ and 1.6 × 10^−5^, respectively). For the average number of eggs per spawn, OGDM binned predictions were not significantly different from experimentally observed values (*p*-value = 0.75). For the average number of spawns per female, the OGDM binned predictions were significantly different at a level of α = 0.05 (*p*-value = 0.04), though the predictions are much closer to the measured data than the OGDM predictions without binning. These results suggest that individual female fathead minnows in a group spawning design spawn in a fashion similar to females in a paired spawning design, and that binning model-predicted eggs spawned by the females in a group on the same day corrects for substantial differences originally observed in unbinned model-predicted eggs per spawn and spawns per female.

Experimentally, the above spawning is rarely observed. An attempt is made to determine whether all the eggs collected from a group came from the same spawn by visually examining eggs under a microscope after they are removed from the spawning tile. If all the eggs are at approximately the same developmental stage, they are assumed to come from the same female and constitute a single spawn. It would be extremely rare to have multiple females spawning with the same male at the same time. Occasionally, there may be eggs that differ developmentally and they are enumerated as two separate spawns. However, it is generally impractical to determine whether they are from the same or different females (e.g., through genotyping or DNA fingerprinting).

### Prediction of steroid synthesis inhibitor impacts on fathead minnow spawning

#### Fadrozole

**Fig 3:**
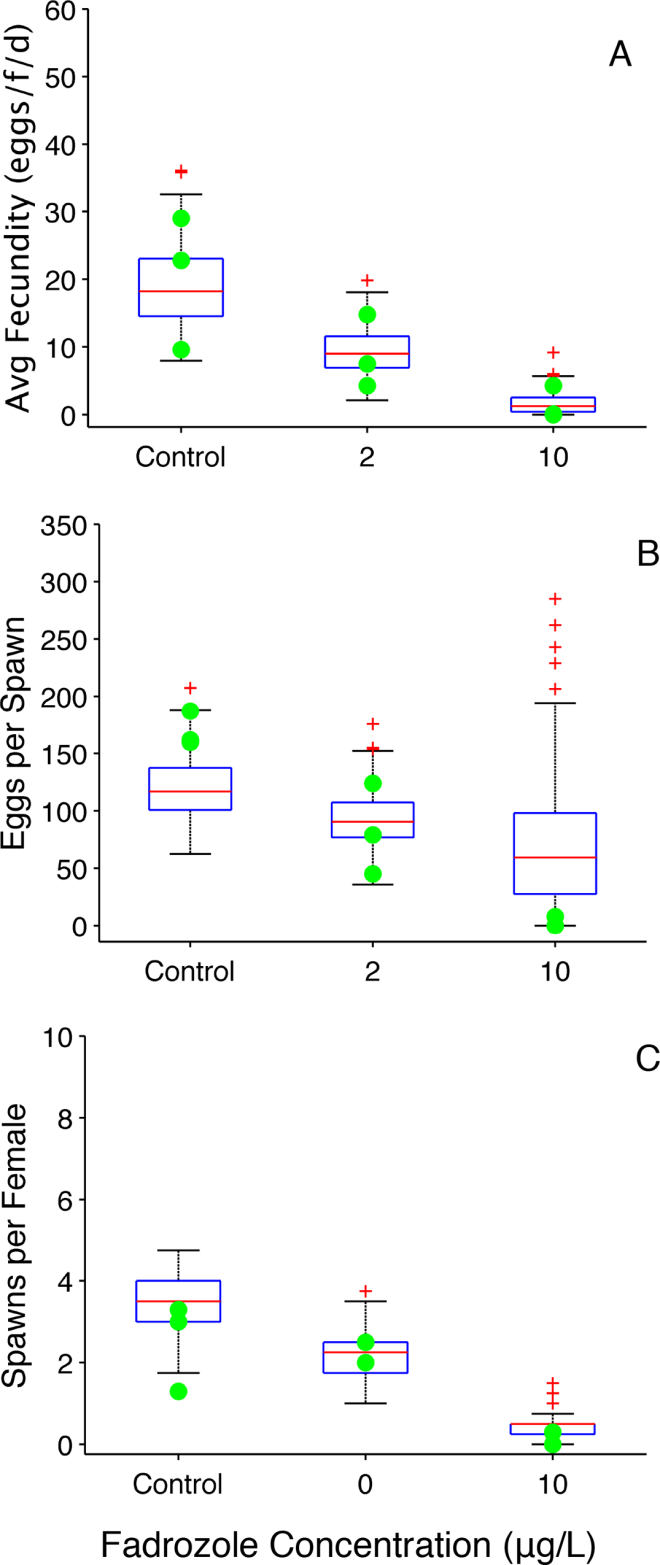
Fadrozole Reproduction Metrics (group spawning design). (A) Average fecundity (eggs•female^-1^•day^-1^). (B) Average number of eggs per spawn. A value of zero indicates that a fish did not spawn during the experiment. (C) Average number of spawns per female. Filled circles represent experimentally observed values [8]. Boxplots represent 150 OGDM-predicted values. In the boxplots, the red line represents the median, lower and upper edges of the box are the 25^th^ and 75^th^ percentiles, respectively, lower and upper whiskers denote the most extreme values that are not outliers (~2.7σ or 99.3 percentile for normally distributed values), and the red + symbol represents outliers.

**Fig 4:**
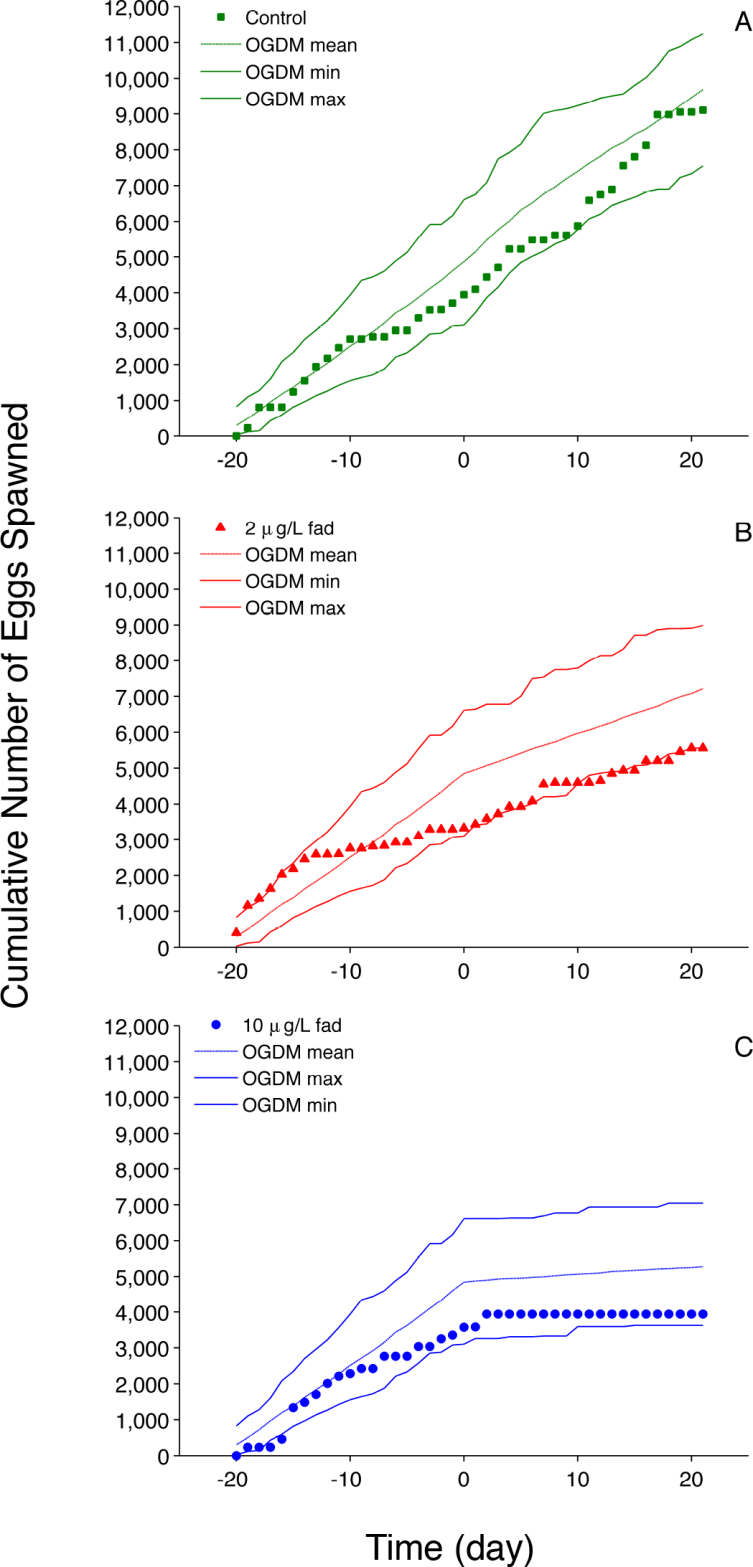
Fadrozole (fad) Cumulative Fecundity. Pre-exposure days reported as negative values; exposure began on day 0. (A) control (0 μg fad•L^-1^); (B) 2 μg fad•L^-1^ exposure; (C) 10 μg fad•L^-1^ exposure. Filled markers represent experimental data [8]; dashed lines represent the mean of 50 cumulative fecundity model predictions; solid lines represent the minimum and maximum cumulative fecundity values of 50 OGDM model predictions.

The OGDM predictions of average fecundity (Fig 3A) as well as cumulative fecundity (Fig 4) encompass experimentally observed values for control, 2 μg•L^-1^ and 10 μg•L^-1^ fadrozole exposure concentrations. Reproduction metrics from the 50 μg•L^-1^ exposure were not plotted because only seven of 150 simulated values (50 simulations of three groups of fathead minnows) were greater than zero and ranged from 0.40 to 2.1 eggs•female^-1^•day^-1^. Experimentally observed average fecundity values for the 50 μg•L^-1^ exposure concentration were 1.1, 1.0 and 1.5 eggs•female^-1^•day^-1^.

OGDM predictions of the average number of eggs per spawn (Fig 3B) based upon binning spawns on the same day encompass experimentally observed values for the control and 2 μg•L^-1^ exposure concentration. For the 10 μg•L^-1^ exposure concentration model predictions show greater variability compared to the other two fadrozole treatments, which is consistent with the experimental values. Without binning spawns on the same day, model predictions of eggs per spawn and spawns per female for control fish were most affected (see S1 Fig): for eggs per spawn, all model predictions are lower than the experimentally observed values, and for spawns per female, the lower 25^th^ percentile overlapped with experimentally obtained values, but the binned predictions encompass the data better

The average number of spawns per female (Fig 3C) is predicted well by the OGDM. One experimentally observed value (= 1.3) for a control group is approximately 25% lower than the lowest OGDM prediction for controls (minimum = 1.75). As observed previously, binning spawns on the same day improves OGDM predictions of the average number of eggs per spawn and average number of spawns per female. For all exposures simulated, the OGDM does a good job of predicting experimentally observed results and capturing the variability in those results.

#### Ketoconazole

**Fig 5:**
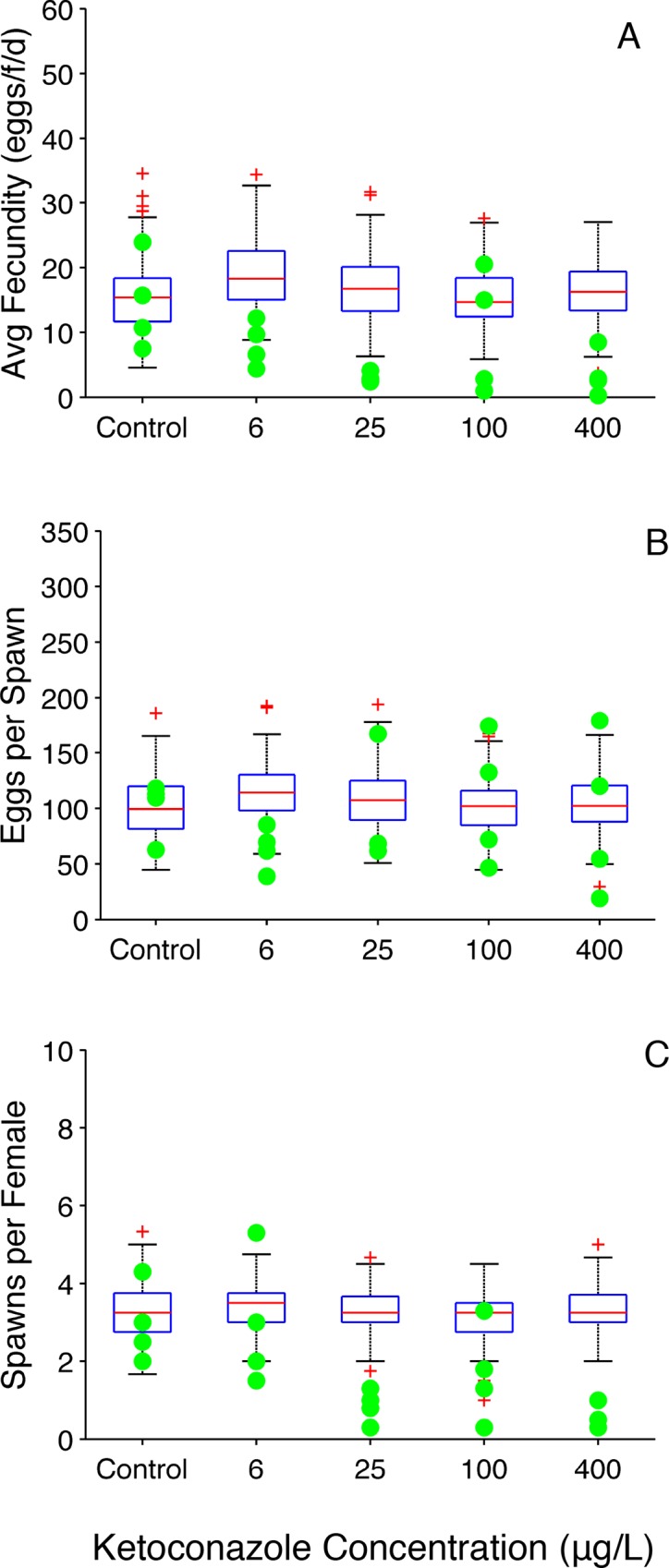
Ketoconazole Reproduction Metrics (group spawning design). (A) Average fecundity (eggs•female^-1^•day^-1^). (B) Average number of eggs per spawn. A value of zero indicates that a fish did not spawn during the experiment. (C) Average number of spawns per female. Filled circles represent experimentally observed values [12]. Boxplots represent 200 OGDM-predicted values. In the boxplots, the red line represents the median, lower and upper edges of the box are the 25^th^ and 75^th^ percentiles, respectively, lower and upper whiskers denote the most extreme values that are not outliers (~2.7σ or 99.3 percentile for normally distributed values), and the red + symbol represents outliers.

The OGDM does an exceptional job of predicting all three reproduction metrics for the control fish from the ketoconazole study, including the variability in the experimentally observed values (Fig 5). In ketoconazole-exposed fish, model-predictions of average fecundity were generally higher than experimentally observed values. Model predictions of eggs per spawn captured the majority of experimentally observed values, and spawns per female are generally higher than experimentally observed values, suggesting that the larger number of predicted spawns per female is responsible for average fecundity being higher than experimentally observed values.

**Fig 6:**
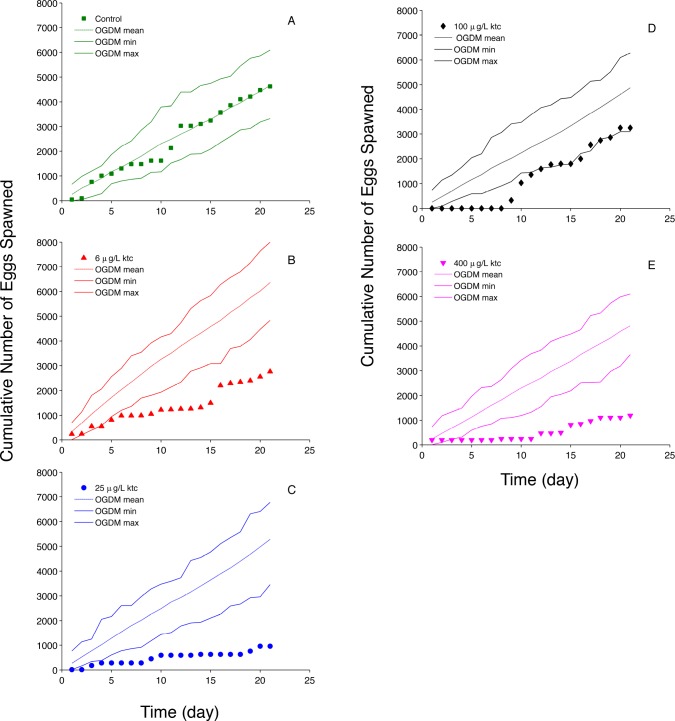
Ketoconazole (ktc) Cumulative Fecundity. (A) control (0 μg ktc•L^-1^); (B) 6 μg ktc•L^-1^; (C) 25 μg ktc •L^-1^; (D) 100 μg ktc•L^-1^; and (E) 400 μg ktc•L^-1^ exposure. Filled markers represent experimental data [12]; dashed lines represent the mean of 50 cumulative fecundity OGDM predictions; solid lines represent the minimum and maximum cumulative fecundity values of 50 OGDM predictions.

For control fish, OGDM predictions capture the experimentally observed cumulative fecundity (Fig 6). However, for exposed fish, the OGDM predicted cumulative fecundity greater than experimentally observed values. Here, compensatory responses that increased plasma VTG levels after prolonged ketoconazole exposure would result in overprediction of fecundity based on plasma VTG measured at the end of a 21-day study since plasma VTG concentration is the only input variable that changes for each treatment. Ankley et al. [12] noted that there was no strong concentration-response relationship for ketoconazole effects on VTG after 21 days of exposure (Table 2). However, in their seven-day range finding study, Ankley et al. observed a stronger concentration-response relationship between ketoconazole exposure and plasma VTG concentration. In addition, a more recent study by Ankley et al. [32] of fathead minnows exposed to 30 and 300 μg ketoconazole•L^-1^ for eight days with a 16-day depuration period reports a time series of plasma VTG concentrations that initially decrease then start to recover during the exposure period in a concentration-dependent fashion. Lower ketoconazole concentrations resulted in faster recovery of plasma VTG during the exposure period. Fecundity data were not reported by Ankley et al. [32], but the concentration-dependent rate of plasma VTG recovery could explain the observed decrease in average fecundity for some groups with increasing exposure concentrations (Fig 5A) while other groups seem to recover more quickly (e.g., in the 100 μg•L^-1^ treatment there are two groups with average fecundity values that are equivalent to control values). This indicates that simulating time varying plasma VTG concentrations in the OGDM could improve predictions for ketoconazole-exposed fathead minnows, and test the hypothesis that changes in plasma VTG concentrations during an exposure can explain the observed changes in fecundity. Modification to the current model would be required and is beyond the scope of this study.

**Table 2:** Measured plasma VTG concentrations (nmol•μL^-1^)^a^ in fathead minnows exposed to ketoconazole for 21 days.

		Ketoconazole Concentration (μg•L^-1^)				
Replicate	Fish	0	6	25	100	400
**1**	**1**	0.077	0.067	0.065	0.065	0.048
**1**	**2**	0.054	0.083	0.101	0.084	0.072
**1**	**3**	0.165	0.142	0.063	0.119	0.090
**1**	**4**	0.122	0.063	0.066	0.176	0.083
**2**	**1**	0.059	0.152	0.088	0.085	0.086
**2**	**2**	0.062	0.162	0.209	0.077	0.081
**2**	**3**	0.055	0.192	0.073	0.060	0.107
**2**	**4**	0.133	0.101	0.162	0.086	0.083
**3**	**1**	0.106	0.176	0.088	0.025	0.108
**3**	**2**	0.080	0.084	0.088	0.111	0.061
**3**	**3**	0.128	0.078	0.029	0.104	0.027
**3**	**4**		0.065	0.069	0.053	
**4**	**1**	0.059	0.188	0.076	0.052	0.054
**4**	**2**	0.049	0.021	0.066	0.090	0.125
**4**	**3**	0.057	0.096	0.092	0.046	0.166
**4**	**4**		0.080			
**Mean**		0.086	0.109	0.089	0.082	0.085

^a^To convert from nmol•μL^-1^ to mg•mL^-1^, multiply value by 156.

#### Propiconazole

**Fig 7:**
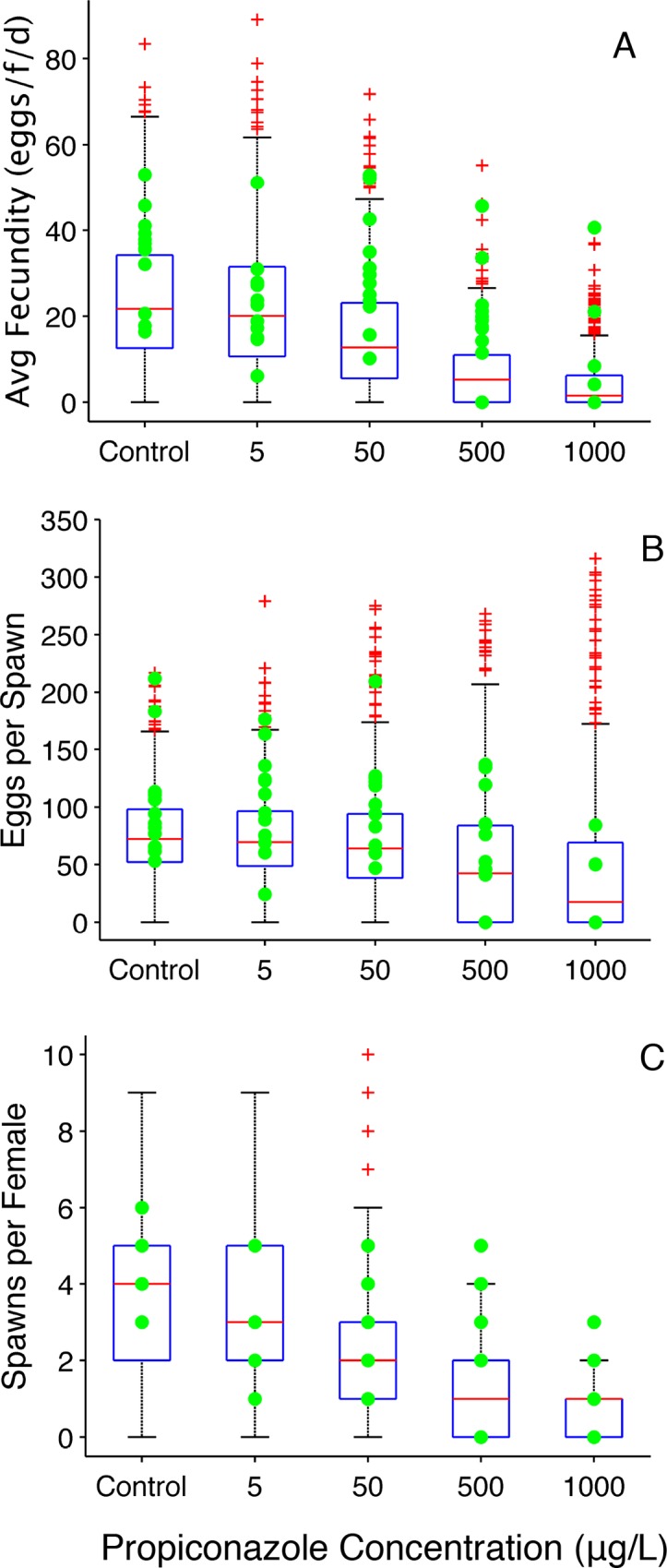
Propiconazole Reproduction Metrics (paired spawning design). (A) Average fecundity (eggs•female^-1^•day^-1^). (B) Average number of eggs per spawn. A value of zero indicates that a fish did not spawn during the experiment. (C) Total number of spawns per female. Filled circles represent experimentally observed values [13]. Boxplots represent 600 OGDM-simulated values. In the boxplots, the red line represents the median, lower and upper edges of the box are the 25^th^ and 75^th^ percentiles, respectively, lower and upper whiskers denote the most extreme values that are not outliers (~2.7σ or 99.3 percentile for normally distributed values), and the red + symbol represents outliers.

Like ketoconazole, propiconazole can inhibit multiple CYPs involved in steroid synthesis. However, unlike ketoconazole the OGDM was able to predict reproduction metrics (Fig 7A–7C) for all concentrations tested in the 21-day reproduction study [13].

**Fig 8:**
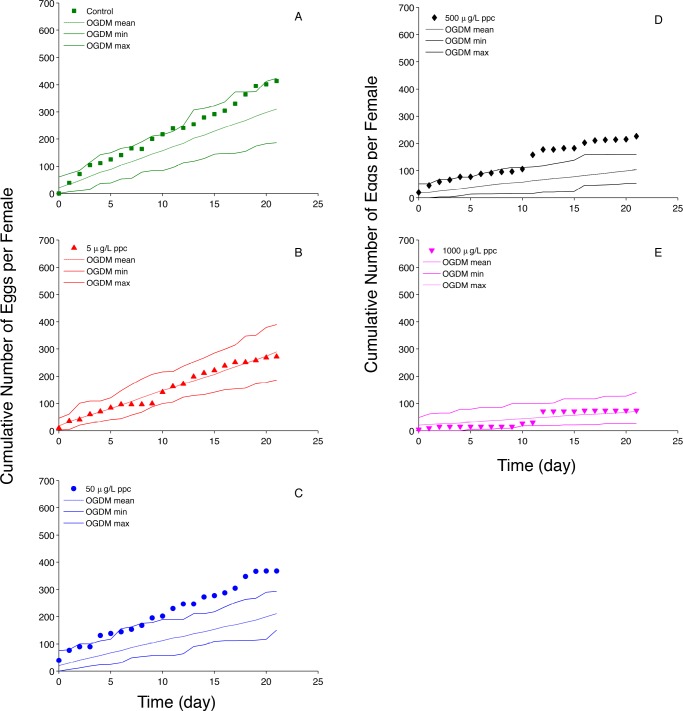
Propiconazole (ppc) Average Cumulative Fecundity. (A) control (0 μg ppc•L^-1^); (B) 5 μg ppc•L^-1^; (C) 50 μg ppc•L^-1^; (D) 500 μg ppc•L^-1^; and (E) 1000 μg ppc•L^-1^. Filled markers represent experimental data (cumulative fecundity divided by the number of females in a treatment group) obtained from figures in Skolness et al. [13]; dashed lines represent the mean of 50 cumulative fecundity OGDM predictions; solid lines represent the minimum and maximum values of individual cumulative fecundity out of 50 OGDM model predictions.

For 50 simulated cohorts in each treatment, the minimum and maximum cumulative fecundity values encompass experimentally observed values for controls (Fig 8A), 5 μg propiconazole•L^-1^ (Fig 8B), and 1000 propiconazole•L^-1^ (Fig 8E). For the 50 and 500 μg propiconazole•L^-1^ exposures, the experimental data were close to the maximum cumulative fecundity predictions until approximately day 10, then the model predictions underestimated experimentally observed cumulative number of eggs spawned per female. This is an interesting result that is counterintuitive given how well the model predicted average fecundity (Fig 7A). However, the values represented by the boxplots in Fig 7A are values predicted for individual fish. In Fig 8, the cumulative number of eggs spawned per female is an average over the 12 fish in a simulated cohort, therefore the presence of one high individual value will not automatically yield a high average number of eggs spawned per female. For example, for the 500 μg •L^-1^ exposure, the largest predicted total number of eggs spawned is 662 and the cohort that this individual fish belongs to has a cumulative number of eggs spawned per female equal to 160 (Fig 8D, value at 21 days). The next largest predicted total number of eggs spawned is 554 and the cumulative number of eggs spawned per female for the cohort is 88 because the other 11 fish in the cohort did not spawn enough eggs to increase the average to a value closer to 160. Overall, OGDM model predictions for reproduction metrics calculated at 21 days fit the experimental data well. Predictions of cumulative fecundity, an endpoint that changes with time during the simulation period, often encompassed experimentally observed values, but were not as robust as OGDM predictions of average fecundity, eggs per spawn, and spawns per female.

#### Prochloraz

**Fig 9:**
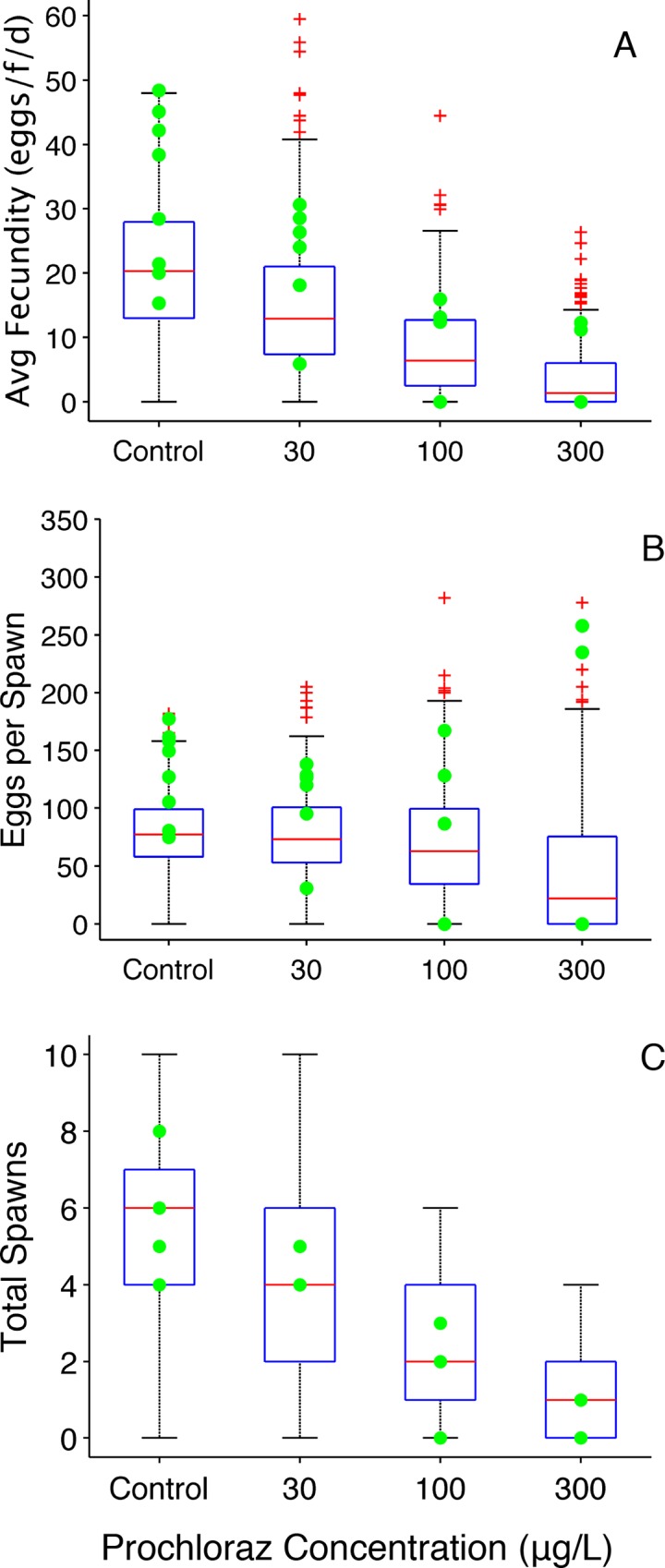
Prochloraz Reproduction Metrics (paired spawning design). (A) Average fecundity (eggs• female^-1^• day^-1^). (B) Average number of eggs per spawn. A value of zero indicates that a fish did not spawn during the experiment. (C) Total number of spawns per female. Filled circles represent experimentally observed values [6]. Boxplots represent 50 OGDM-simulated values for each fish in the treatment: 400 for controls, 300 for 30 μg •L^-1^, 250 for 100 μg •L^-1^, and 300 for μg •L^-1^. In the boxplots, the red line represents the median, lower and upper edges of the box are the 25^th^ and 75^th^ percentiles, respectively, lower and upper whiskers denote the most extreme values that are not outliers (~2.7σ or 99.3 percentile for normally distributed values), and the red + symbol represents outliers.

**Fig 10:**
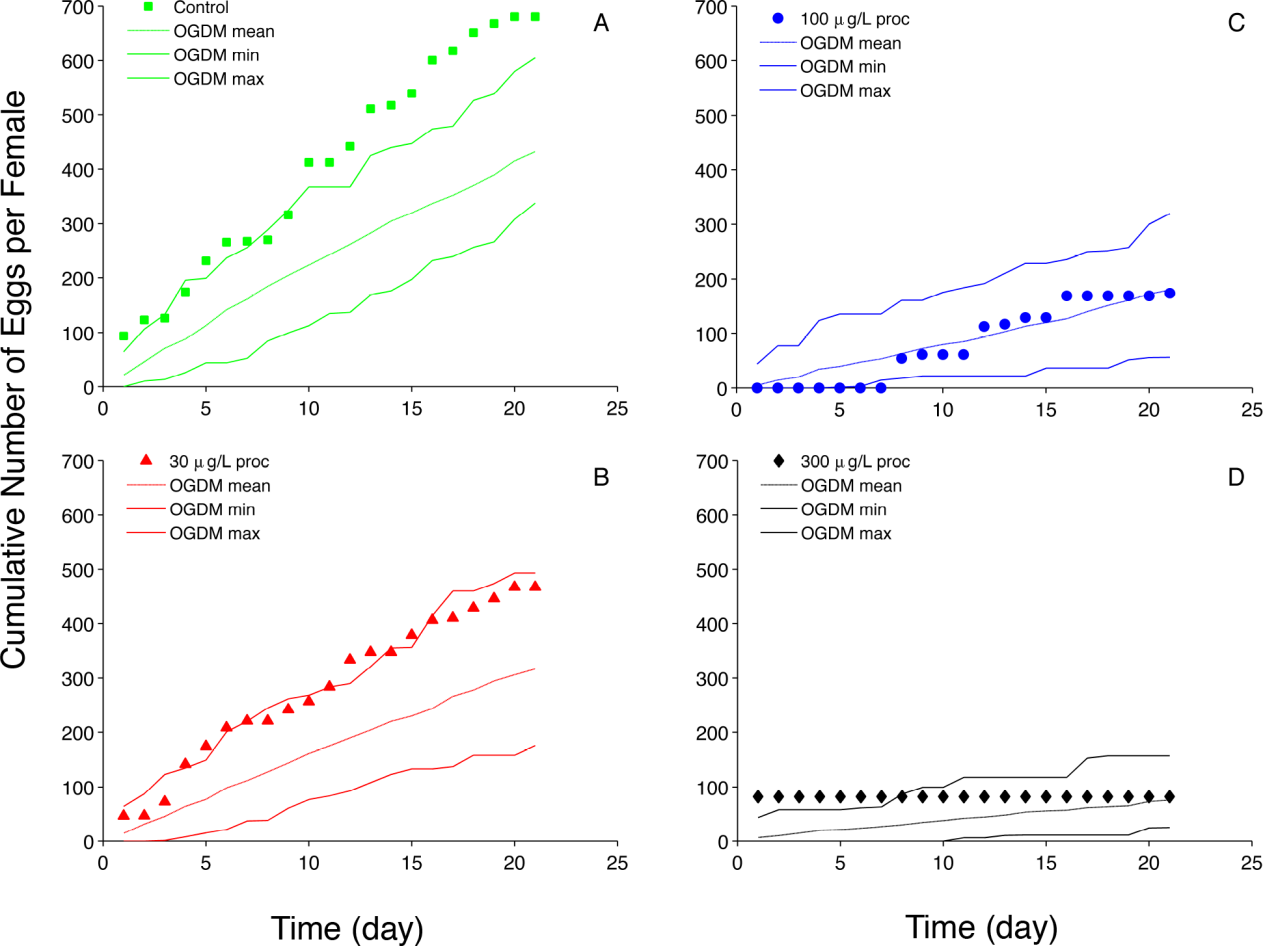
Prochloraz (proc) Average Cumulative Fecundity. (A) control (0 μg proc•L^-1^); (B) 30 μg proc•L^-1^; (C) 100 μg proc•L^-1^; (D) μg proc•L^-1^. Filled markers represent experimental data (cumulative fecundity divided by the number of females in a treatment group) [6]; dashed lines represent the mean of 50 cumulative fecundity model predictions; solid lines represent the minimum and maximum values of individual cumulative fecundity out of 50 OGDM model predictions.

Simulation results for prochloraz are shown in Figs 9 and 10. Simulated prochloraz reproduction metrics encompass the experimentally data reasonably well. There were four fish at the highest exposure concentration (300 μg•L^-1^) that did not spawn during the exposure; two fish spawned once on the first day of the exposure. The boxplot for this treatment (Fig 9B) shows the two fish that spawned in the outlier region, and predicts low values including ‘zero’. The OGDM predicts all reproduction metrics well and does a fairly good job of predicting average cumulative fecundity for all treatment groups. Average cumulative fecundity (Fig 10A) for control fathead minnows is slightly higher than the maximum OGDM prediction for most of the 21-day period, but the slope of the experimental data is similar to that of the OGDM predictions. Thus, if the control fish had not spawned a total of 749 eggs (94 eggs•female^-1^) on the first day, the observed values would be close to the maximum OGDM-predicted values.

#### Fenarimol and Trilostane

OGDM prediction of reproduction metrics for fenarimol (S3A Fig) encompass the observed data well. At the highest test concentration of fenarimol (1000 μg •L^-1^), no spawning occurred, and the OGDM predicted no eggs and spawns for 19 of 200 simulations. Thus, while the OGDM predicts that no spawning will occur, in general the model overpredicts fecundity for this test concentration based upon measured plasma VTG values. At a concentration of 100 μg fenarimol •L^-1^, the OGDM predicts reproduction metrics and average cumulative fecundity (S3B Fig) well.

Trilostane reproduction metrics (S4A Fig) and average cumulative fecundity (S4B Fig) were predicted well by the OGDM at all treatment levels. The experimentally observed average number of spawns per female for 60 and 300 μg trilostane •L^-1^ (S4A Fig panel C) were at or below the median OGDM-predicted values, but were above the lowest whisker of the boxplot; values below the whisker are considered outliers. For the controls, one fish did not spawn during the experiment and the OGDM predicts one case of no eggs and spawns out of 650 simulated values; these fall in the outlier region of the boxplot.

### Application of the OGDM

Future applications of the OGDM include extension to other fish species in the context of using fecundity predictions as input into a population dynamics model to predict population-level impacts of chemicals. With modification of fish-specific parameters (e.g., distributions of clutch sizes and spawning intervals, oocyte recruitment size and critical volume for spawning), the OGDM could be used to simulate oocyte growth dynamics in other continuous-spawning fish species like the fathead minnow, as well as species that spawn on a more limited basis (e.g., annually). For the latter, an additional minor modification of allowing for time varying plasma VTG concentrations would be required. The desirability of this modification also was highlighted by the ketoconazole simulation results where, due to compensatory changes, VTG production changes over time. A critical reason for choosing open source licensing of the OGDM was to facilitate adaptation to a variety of fishes and stressor scenarios.

Another improvement to the functionality of the OGDM is to incorporate VTG mRNA as an input parameter in place of plasma VTG concentration. This would require modifications to the code to simulate the translation of VTG mRNA into VTG protein, and subsequently how plasma VTG concentrations are affected. In addition, model evaluation would require data sets with measurements of VTG mRNA and spawning; measured plasma VTG concentrations would be valuable for comparison of the data set with current simulation results. Open source licensing of the OGDM will allow investigators to customize the OGDM for their own data sets.

In the context of ecological risk assessment and the paradigm of an adverse outcome pathway [15], evaluating effects upon a population is the ultimate goal. *In vitro* assays are increasingly being used to expedite toxicity tests for prioritizing chemicals [3] and extrapolation of results from *in vitro* assays to effects *in vivo* and upon a population is an area where computational models have a significant role. For example, measured changes in VTG concentrations from an *in vitro* liver slice assay (e.g., see [33]) relative to controls could be used calculate corresponding *in vivo* plasma VTG concentrations for input into the OGDM to obtain predictions of effects upon fecundity.

Although it is possible to experimentally determine chemical effects on fish populations (e.g., see [34]) this is a time-consuming and resource-intensive process. Consequently, population dynamics models typically are used to predict population-level effects based upon changes in fertility and survival rates of different age classes of fish [1]. As noted throughout this paper, the 21-day reproduction studies reported decreases in fecundity without any decrease in hatching success. Thus, while the numbers of eggs spawned were affected by chemical treatments, egg fertility was unaffected though the number of offspring was reduced relative to control fish. For population dynamics modeling, fecundity is a good surrogate metric of fertility, and the OGDM can be used to link measured or model-predicted changes in plasma VTG [35–37] with effects upon a population.

## Conclusions

We showed that the OGDM produces results consistent with the MCSim oocyte growth dynamics model for fathead minnows exposed to the androgenic chemical 17β-trenbolone, the only chemical treatment simulated by Li et al [16]. It also robustly predicts the fecundity of control fish and the impact of five steroidogenesis inhibitors (fadrozole, propiconazole, prochloraz, fenarimol and trilostane) on fathead minnow reproduction metrics. The model partially predicted impacts of a sixth steroidogenesis inhibitor, ketoconazole. Some OGDM average fecundity predictions were in the range of experimentally observed reproduction metrics for ketoconazole-exposed fathead minnows but, in general, the OGDM over-predicted average fecundity likely due to compensatory responses in plasma VTG in the fish that were not accounted for in the simulation. We expect that simulation of time varying plasma VTG concentrations would improve OGDM predictions for ketoconazole treated fathead minnows.

Our simulation results reinforce empirical observations concerning the utility of plasma VTG concentration as a good predictor of fish fecundity (e.g., see [5,38]). Given the role of plasma VTG as a key event in multiple adverse outcome pathways leading to reproductive dysfunction in female fish, the OGDM should serve as useful tool for predictive ecotoxicology [39]. As shown, the OGDM predicts average fecundity robustly for both group and paired spawning design studies using the 21-day fathead minnow assay. For the average number of eggs per spawn and the average number of spawns per female, binning the eggs spawned on the same day by females in a group improves OGDM predictions of these endpoints when compared to experimentally observed results. This indicates that female fathead minnows in group-spawning design studies spawn similarly to females in paired spawning design studies.

Overall, the OGDM performed well in our model evaluations. Benefits of the OGDM over a purely statistical model are its biological underpinning and its ability to predict population variability in reproduction metrics. With a user-friendly interface, platform independence, and open source licensing, the OGDM can be used to test a wide variety of scenarios exploring how stressors that affect vitellogenin production will impact fecundity.

## Supporting Information

S1 Appendix OGDM user’s guide. (PDF)

S1 Fig Fadrozole reproduction metrics (group spawning design)—no spawn binning. (PDF)

S2 Fig Ketoconazole reproduction metrics (group spawning design)—no spawn binning. (PDF)

S3 Fig Fenarimol OGDM predictions: (A) reproduction metrics; (B) average cumulative fecundity. (PDF)

S4 Fig Trilostane OGDM predictions: (A) reproduction metrics; (B) average cumulative fecundity. (PDF)

S1 Table Data for control fathead minnows. (PDF)
