# Supplementary material for: Predicting Fecundity of Fathead Minnows (Pimephales promelas) Exposed to Endocrine-Disrupting Chemicals Using a MATLAB®-Based Model of Oocyte Growth Dynamics
Source: PLoS One. 2016 Jan 12;11(1):e0146594. doi: 10.1371/journal.pone.0146594 (PMC4710531; doi:10.1371/journal.pone.0146594)
Supplement: S1 Appendix — (PDF) [file pone.0146594.s001.pdf]

OGDM User's Guide

Graphical User Interface (GUI)

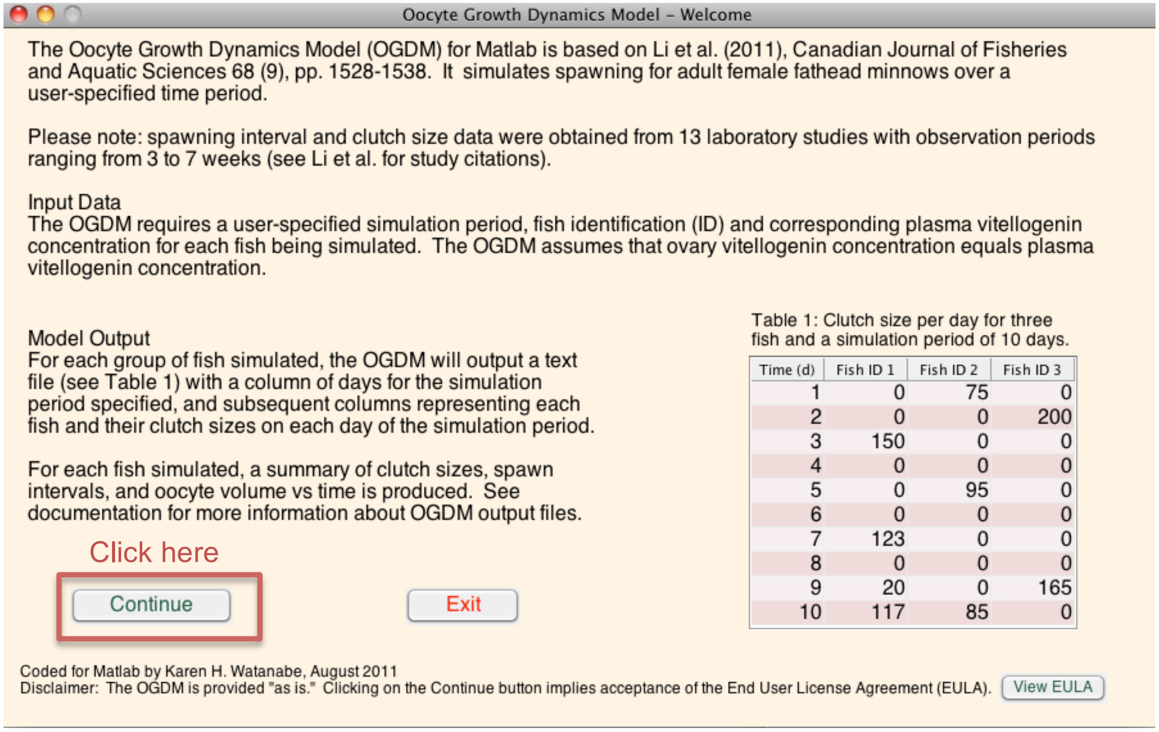

Figure 1. OGDM welcome window

When starting the Matlab® version of the oocyte growth dynamic model (OGDM) the first window that you will see is shown in Figure 1. Clicking on the Continue button lead to the next window (Figure 2).

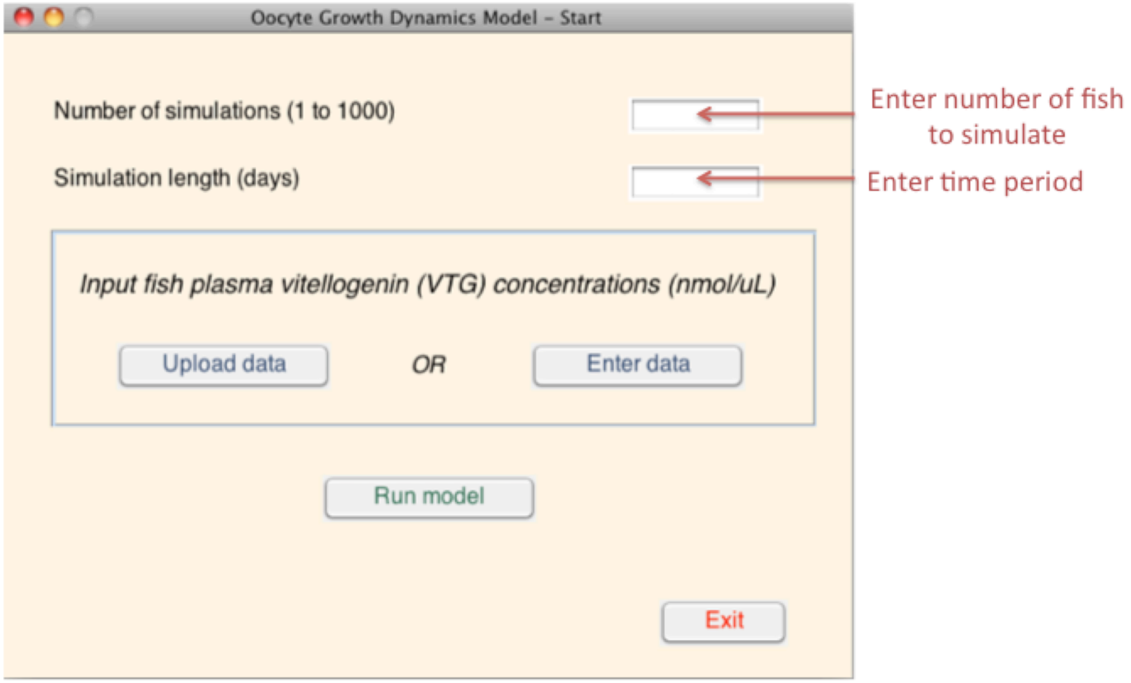

Figure 2. Main OGDM dialog box

This is the main interface for running the OGDM. This window allows users to enter input data (i.e., # fish simulations, simulation length, and plasma vitellogenin concentrations), run the model and exit the OGDM. After entering the number of simulations and the simulation length, either 'Upload data' or 'Enter data' should be chosen.

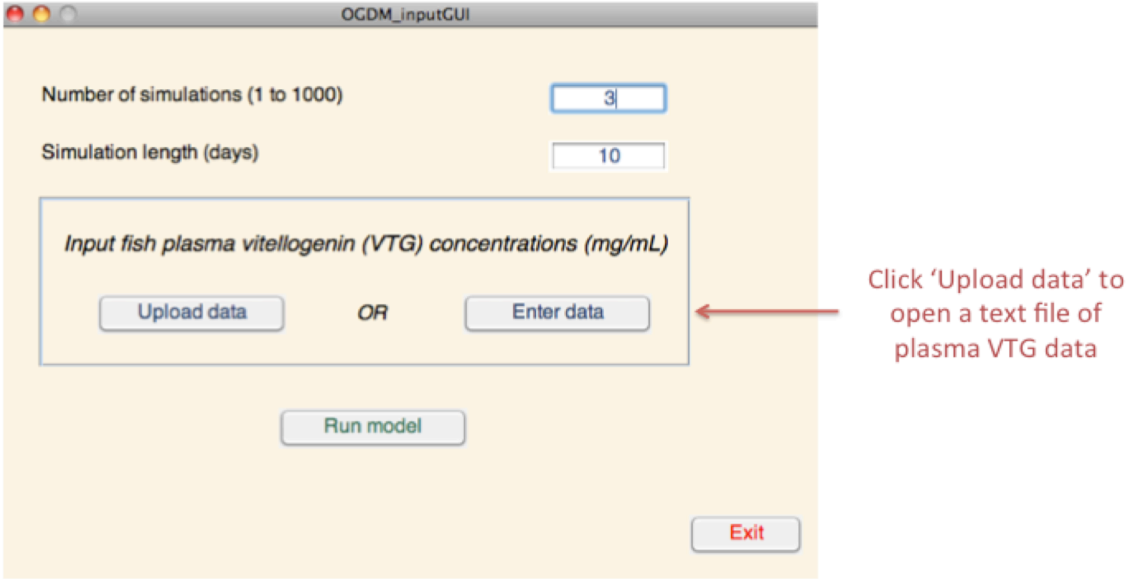

Figure 3. Data entry

Clicking on the 'Upload data' button opens the following dialog box.

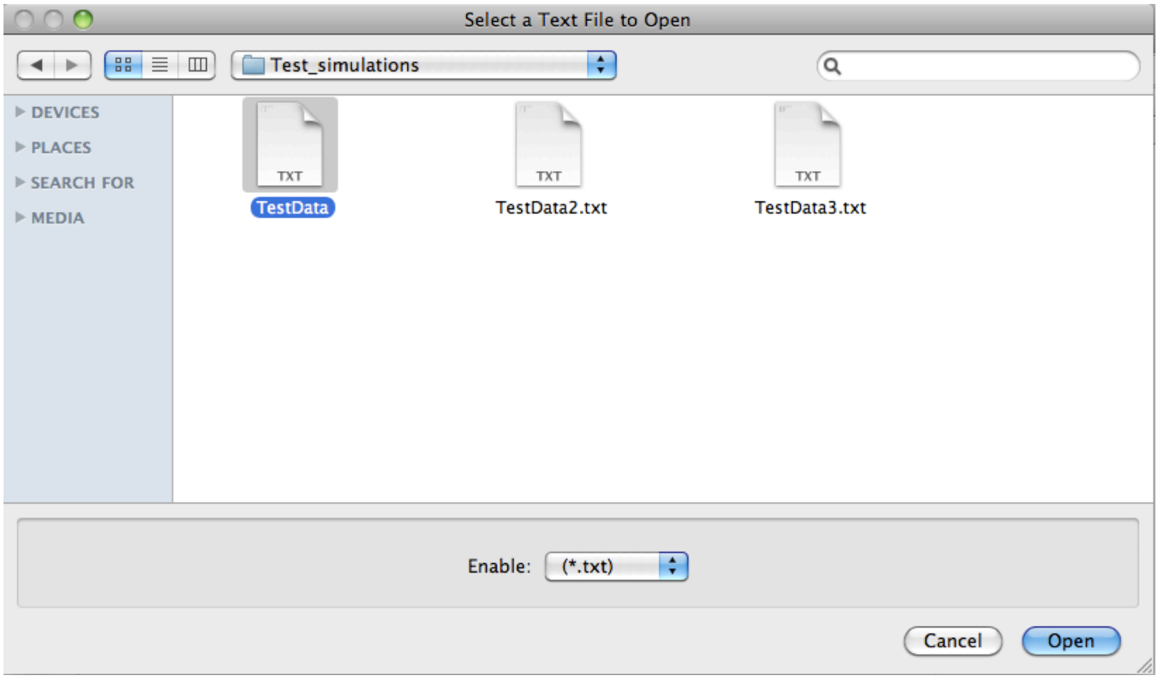

Figure 4. Dialog box to select a file.

Select a text file containing fish IDs (alphanumeric entries okay) in column 1 and plasma vitellogenin concentrations (nmol•µL^{-1}) in column 2; row 1 should be a header line. A maximum of 1000 fish may be simulated at a time.

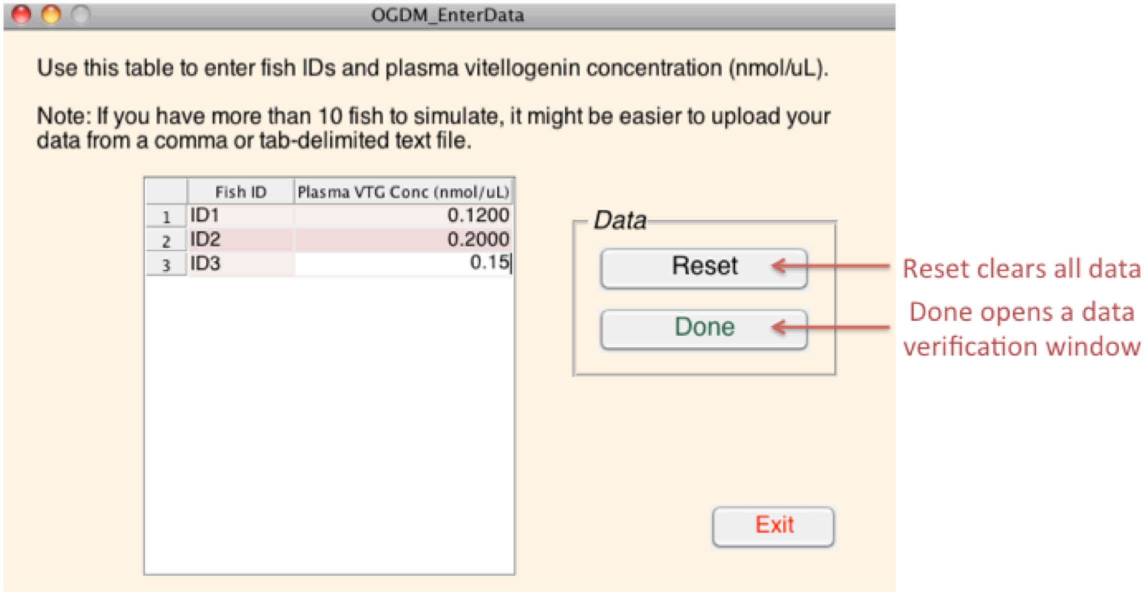

Figure 5. Manual data entry.

An alternative to uploading plasma vitellogenin concentrations from an input file is to manually enter data as shown in Figure 5. Clicking on 'Done' opens the window below to verify that the values entered are correct.

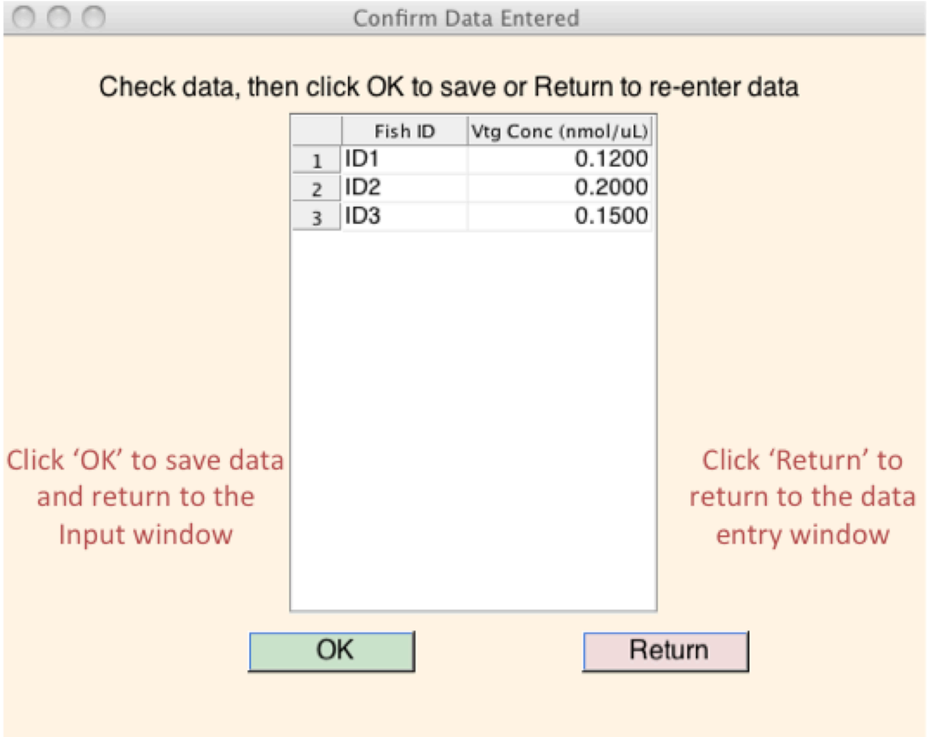

Figure 6. Confirm data entered manually or uploaded from a text file.

After clicking the ‘OK’ button in Figure 6, the OGDM returns to the window shown in Figure 2. Clicking on ‘Run model’ starts the model simulation(s). 600 simulations lasting 21 days takes less than five minutes on a 2011 MacBook Pro. Run times will vary.

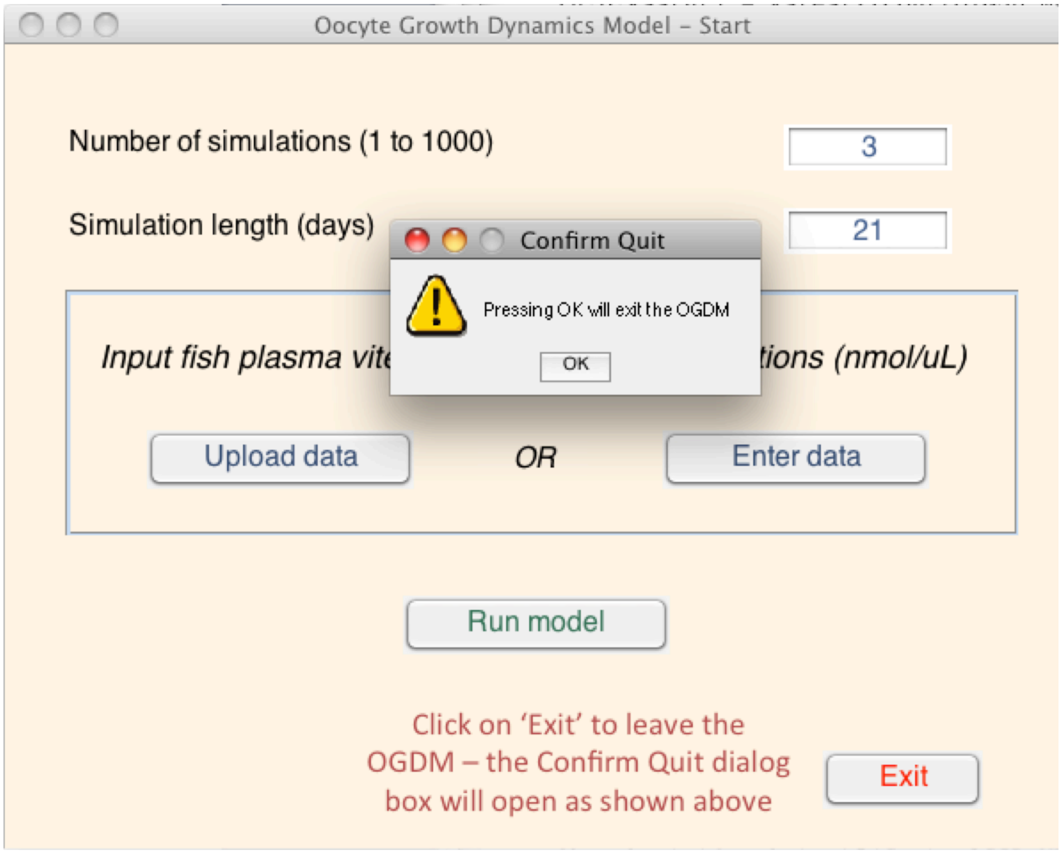

Figure 7. Exit OGDM

To close/quit the OGDM, click on the ‘Exit’ button at any time. A confirm quit dialog box will appear.

Sample OGDM Files

Plasma vitellogenin input file

MED21d_fad0_VtgConcData_100cohorts.txt

Fish ID	Vtg_nmol/uL
3	0.045955128
4	0.084538462
5	0.090230769
6	0.054442308
9	0.12774359
10	0.245897436
11	0.145089744
12	0.237346154
15	0.082807692
16	0.252089744
17	0.10224359
18	0.033134615
3	0.045955128
4	0.084538462
5	0.090230769
6	0.054442308
9	0.12774359
10	0.245897436
11	0.145089744
12	0.237346154
15	0.082807692
16	0.252089744
17	0.10224359
18	0.033134615
3	0.045955128
4	0.084538462
5	0.090230769
6	0.054442308
9	0.12774359

Figure 8. Plasma vitellogenin input file for upload into OGDM.

The file shown here was used to simulate fadrozole control fish. Note that fish IDs repeat in order to simulate one fish more than once. The file should have one header line, i.e., data should start on line 2.

OGDM output files

	A	B	C	D	E	F
1	Fish simulation: # 1	Fish ID: ID1	Plasma Vtg Conc simulated (nmol/uL) =	0.12		
2	Total number of eggs spawned =	1445	Total eggs spawned in 21 days =	389		
3	Total number of spawns =	15	Spawns in 21 days =	5		
4						
5	Recruit Time (d)	Spawn Time (d)	Growth Time (d)	Spawn Interval (d)	Batch size	Clutch Size
6	-76	-64	12	12	34	34
7	-67	-54	13	10	98	98
8	-61	-49	12	5	124	124
9	-57	-45	12	4	315	315
10	-56	-43	13	2	51	51
11	-53	-40	13	3	8	8
12	-49	-36	13	4	27	27
13	-33	-20	13	16	224	224
14	-31	-18	13	2	22	22
15	-12	0	12	18	153	153
16	-7	5	12	5	69	69
17	-4	7	11	2	242	242
18	1	12	11	5	9	9
19	4	15	11	3	50	50
20	7	18	11	3	19	19
21	15	0	0	0	135	0
22						

Figure 9. ResultsSummary_*.csv

This file contains a summary of OGDM results for one fish. It includes the fish ID and the plasma VTG concentration that was used in the simulation in addition to detailed spawning predictions.

Recruit time is the day that a batch of oogonia are recruited into the growth and development phase. Spawn time is the day that the batch of oocytes was spawned. Growth time is the number of days that it took a batch of oogonia to reach the critical volume for spawning (=0.52 μL). Spawn interval is the number of days between successive spawns. Batch size is the number of eggs recruited into growth and development. Clutch size is equal to the batch size as long as oocytes in a batch reach the critical volume for spawning during the simulation period.

	A	B	C	D
1	Time (d)	ID1	ID2	ID3
2	-80	0	0	0
3	-79	0	0	0
4	-78	0	0	0
5	-77	0	0	0
6	-76	0	0	0
7	-75	0	0	0
8	-74	0	0	0
9	-73	0	0	0
10	-72	0	0	0
11	-71	0	0	0
12	-70	0	0	0
13	-69	0	0	0
14	-68	0	0	0
15	-67	0	0	0
16	-66	0	0	0
17	-65	0	0	0
18	-64	34	0	0
19	-63	0	0	0
20	-62	0	0	0
21	-61	0	40	0
22	-60	0	0	0
23	-59	0	52	0
24	-58	0	0	0
25	-57	0	0	0
26	-56	0	79	49

	A	B	C	D
1	Time (d)	ID1	ID2	ID3
83	1	0	87	0
84	2	0	0	92
85	3	0	56	23
86	4	0	284	150
87	5	69	67	0
88	6	0	63	0
89	7	242	30	0
90	8	0	59	130
91	9	0	0	0
92	10	0	0	0
93	11	0	0	26
94	12	9	0	0
95	13	0	0	0
96	14	0	76	83
97	15	50	0	0
98	16	0	0	0
99	17	0	0	0
100	18	19	92	0
101	19	0	0	0
102	20	0	0	36
103	21	0	0	0
104				
105				
106				
107				
108				

Figure 10. OGDM_ClutchSizeSummary.txt

This file contains all clutches spawned by each fish simulated (up to 1000 in one run of the model). It starts at t = -80 days and ends with the user-specified simulation length (days). OGDM_ClutchSizeSummary.txt was imported into Excel to display it here.

	A	B	C	D	E
1	Simulation#	# Spawns	Total Eggs Spawned	Eggs Spawned in 21 days	# Spawns in 21 days
2	1	15	1445	389	5
3	2	28	1989	814	9
4	3	23	1499	540	7
5					
6					
7					

Figure 11. OGDM_SpawningSummary.txt

Columns B and C report results for the entire simulation period (-81 days + simulation length (days) specified by the user). Columns D and E report results for just the user-specified simulation period.
