# Supplementary material for: Predicting Fecundity of Fathead Minnows (Pimephales promelas) Exposed to Endocrine-Disrupting Chemicals Using a MATLAB®-Based Model of Oocyte Growth Dynamics
Source: PLoS One. 2016 Jan 12;11(1):e0146594. doi: 10.1371/journal.pone.0146594 (PMC4710531; doi:10.1371/journal.pone.0146594)
Supplement: S1 Fig — (PDF) [file pone.0146594.s002.pdf]

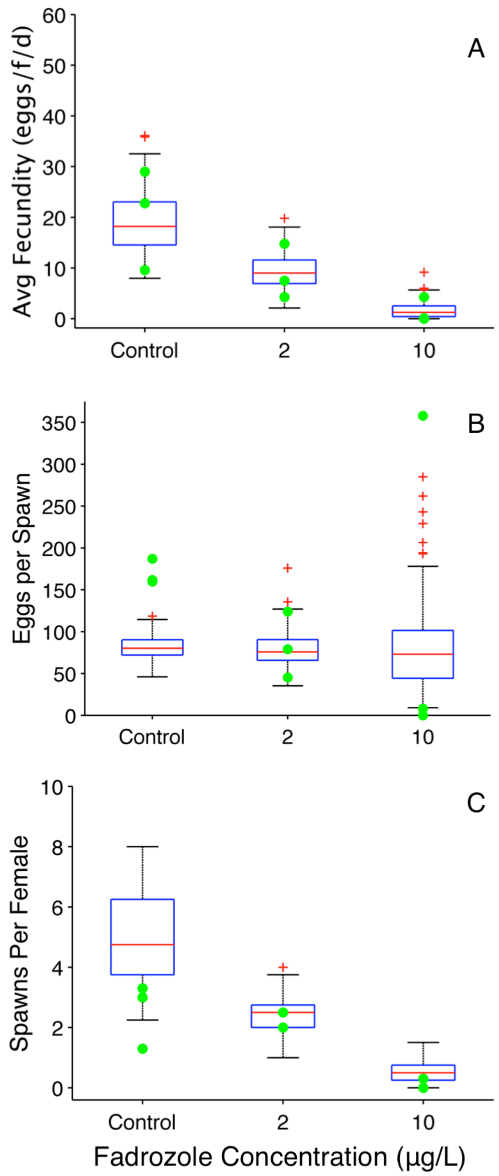

S1 Fig. Fadrozole reproduction metrics (group spawning design) - no spawn binning.
A – Average fecundity (eggs•female^{-1}•day^{-1}). B – Average number of eggs per spawn. A value of zero indicates that a fish did not spawn during the experiment. C – Average number of spawns per female. Filled circles represent experimentally observed values [1]. Boxplots represent 50 OGDM-simulated values. In the boxplots, the red line represents the median, lower and upper edges of the box are the 25th and 75th percentiles, respectively, lower and upper whiskers denote the most extreme values that are not outliers (~2.7σ or 99.3 percentile for normally distributed values), and the red + symbol represents outliers.

References cited:

1. Ankley GT, Kahl MD, Jensen KM, Hornung MW, Korte JJ, et al. (2002) Evaluation of the aromatase inhibitor fadrozole in a short-term reproduction assay with the fathead minnow (*Pimephales promelas*). Toxicological Sciences 67: 121-130.
