# Supplementary material for: Predicting Fecundity of Fathead Minnows (Pimephales promelas) Exposed to Endocrine-Disrupting Chemicals Using a MATLAB®-Based Model of Oocyte Growth Dynamics
Source: PLoS One. 2016 Jan 12;11(1):e0146594. doi: 10.1371/journal.pone.0146594 (PMC4710531; doi:10.1371/journal.pone.0146594)
Supplement: S2 Fig — (PDF) [file pone.0146594.s003.pdf]

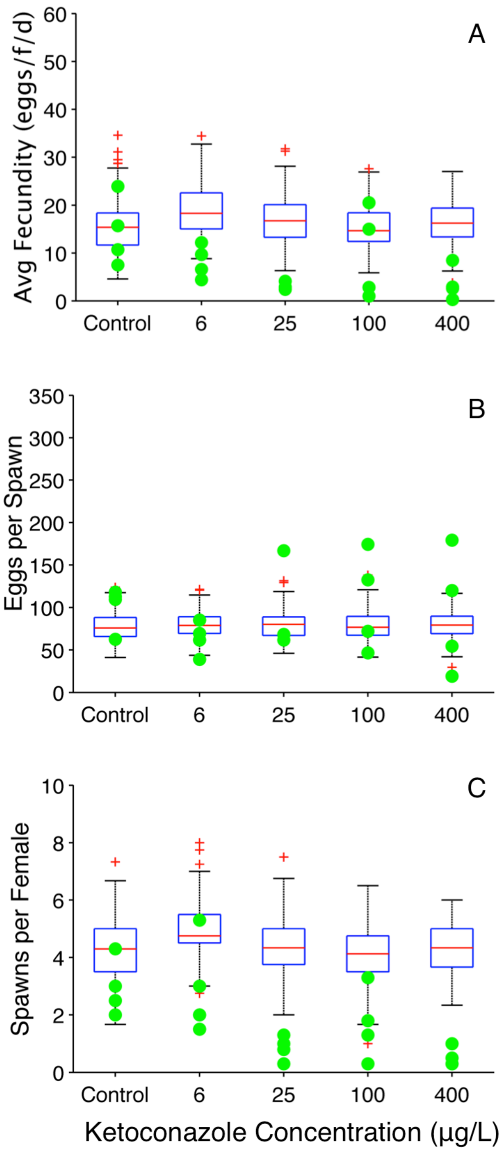

S2 Fig. Ketoconazole reproduction metrics (group spawning design) - no spawn binning.
A – Average fecundity ($eggs \bullet female^{-1} \bullet day^{-1}$). B - Average number of eggs per spawn. C – Average number of spawns per female. Filled circles represent experimentally observed values [1]. Boxplots represent 50 OGDM-simulated values. In the boxplots, the red line represents the median, lower and upper edges of the box are the 25th and 75th percentiles, respectively, lower and upper whiskers denote the most extreme values that are not outliers (~2.7σ or 99.3 percentile for normally distributed values), and the red + symbol represents outliers.

References cited:

1. Ankley GT, Jensen KM, Kahl MD, Makynen EA, Blake LS, et al. (2007) Ketoconazole in the fathead minnow (*Pimephales promelas*): reproductive toxicity and biological compensation. Environmental Toxicology and Chemistry 26: 1214-1223.
