# Supplementary material for: Predicting Fecundity of Fathead Minnows (Pimephales promelas) Exposed to Endocrine-Disrupting Chemicals Using a MATLAB®-Based Model of Oocyte Growth Dynamics
Source: PLoS One. 2016 Jan 12;11(1):e0146594. doi: 10.1371/journal.pone.0146594 (PMC4710531; doi:10.1371/journal.pone.0146594)
Supplement: S3 Fig — (PDF) [file pone.0146594.s004.pdf]

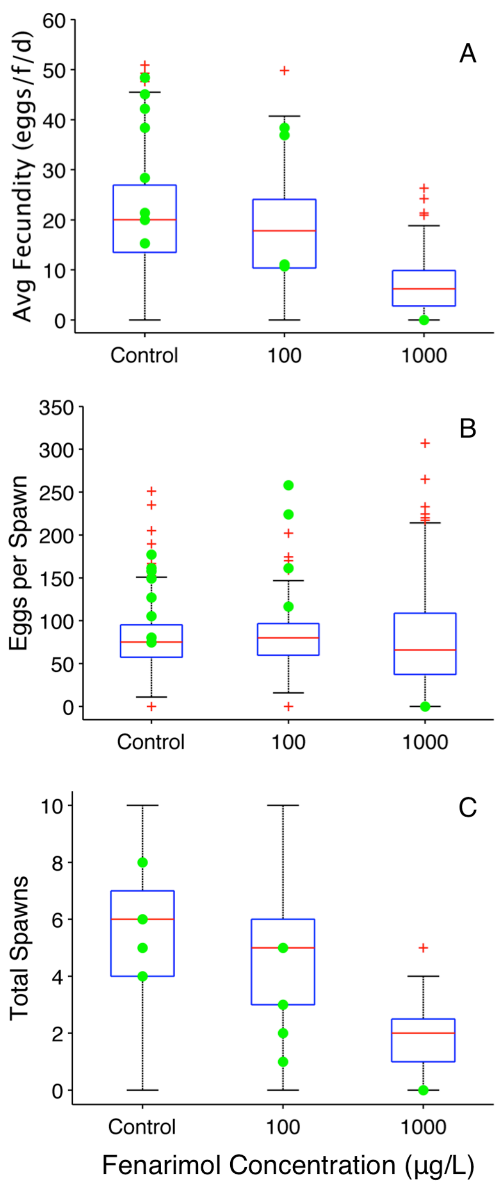

S3A Fig. Fenarimol Reproduction Metrics (paired spawning design).

A – Average fecundity (eggs•female^{-1}• day^{-1}). B - Average number of eggs per spawn. A value of zero indicates that a fish did not spawn during the experiment. C – Total number of spawns per female. Filled circles represent experimentally observed values [1]. Boxplots represent 50 OGDM-simulated values for each fish in the treatment: 400 for controls, 200 for 100 and 1000 µg • L^{-1}. In the boxplots, the red line represents the median, lower and upper edges of the box are the 25th and 75th percentiles, respectively, lower and upper whiskers denote the most extreme values that are not outliers (~2.7σ or 99.3 percentile for normally distributed values), and the red + symbol represents outliers.

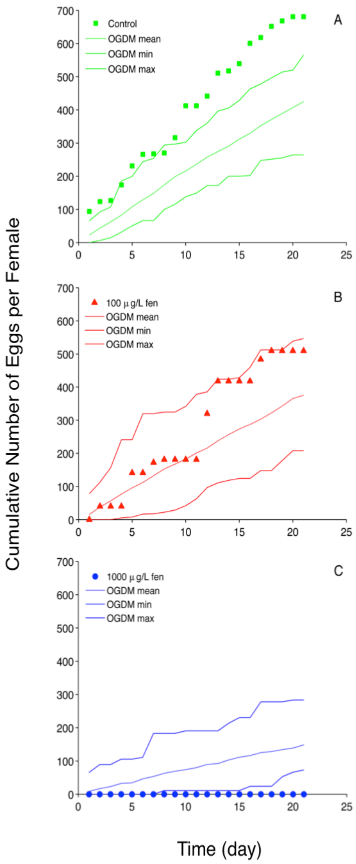

S3B Fig. Femarimol (fen) Cumulative Number of Eggs per Female.
A – control (0 µg fen•L^{-1}); B – 100 µg fen•L^{-1}; C – 1000 µg fen•L^{-1}. Filled markers represent experimental data (cumulative fecundity divided by the number of females in a treatment group) [1]; dashed lines represent the mean of 50 cumulative fecundity model predictions; solid lines represent the minimum and maximum values of individual cumulative fecundity out of 50 OGDM model predictions.

References cited:

1. Ankley GT, Jensen KM, Durhan EJ, Makynen EA, Butterworth BC, et al. (2005) Effects of two fungicides with multiple modes of action on reproductive endocrine function in the fathead minnow (*Pimephales promelas*). Toxicological Sciences 86: 300-308.
