# Supplementary material for: Predicting Fecundity of Fathead Minnows (Pimephales promelas) Exposed to Endocrine-Disrupting Chemicals Using a MATLAB®-Based Model of Oocyte Growth Dynamics
Source: PLoS One. 2016 Jan 12;11(1):e0146594. doi: 10.1371/journal.pone.0146594 (PMC4710531; doi:10.1371/journal.pone.0146594)
Supplement: S1 Table — (PDF) [file pone.0146594.s006.pdf]

S1 Table. Data from 21-day reproduction studies that used a group spawning design. Plasma vitellogenin (VTG) concentrations and fecundity metrics for control fish.

Fish ID	Plasma VTG ($mg \bullet mL^{-1}$)	Plasma VTG ($nmol \bullet \mu L^{-1}$)	Average fecundity ($eggs \bullet female^{-1} \bullet day^{-1}$)	Spawns per Female	Eggs per Spawn	Reference
bTren_3	19.27	0.124	11.5	ND	ND	[1]
bTren_4	33.27	0.213				
bTren_5	22.67	0.145				
bTren_6	23.33	0.150				
bTren_9	25.20	0.162	15.5	ND	ND	
bTren_10	36.26	0.232				
bTren_11	38.12	0.244				
bTren_12	27.17	0.174				
bTren_15	26.46	0.170	9.8	ND	ND	
bTren_16	28.97	0.186				
bTren_17	19.67	0.126				
bTren_18	23.05	0.148				
fad_3	7.17	0.046	22.8	3.0	159.8	[2]
fad_4	13.19	0.085				
fad_5	14.08	0.090				
fad_6	8.49	0.054				
fad_9	19.93	0.128	29.0	3.3	187.1	
fad_10	38.36	0.246				
fad_11	22.63	0.145				
fad_12	37.03	0.237				
fad_15	12.92	0.083	9.6	1.3	161.8	
fad_16	39.33	0.252				
fad_17	15.95	0.102				
fad_18	5.17	0.033				
flut_51	14.64	0.094	11.3	2.8	86.4	[3]
flut_52	17.43	0.112				
flut_53	17.58	0.113				
flut_54	22.00	0.141				
flut_27	18.83	0.121	26.9	3.3	173.8	
flut_28	18.59	0.119				
flut_29	12.57	0.081				
flut_30	12.95	0.083				
methoxy_9	16.93	0.109	20.7	3.8	116.0	[4]
methoxy_10	7.34	0.047				
methoxy_11	17.47	0.112				
methoxy_12	7.10	0.046				
methoxy_15	15.97	0.102	20.2	3.8	113.0	
methoxy_16	15.82	0.101				
methoxy_17	10.48	0.067				
methoxy_18	13.30	0.085				
methoxy_21	12.63	0.081	20.5	2.3	191.0	[4]
methoxy_22	14.56	0.093				
methoxy_23	16.57	0.106				
methoxy_24	10.76	0.069				

**Fish ID**	**Plasma VTG ($mg\bullet mL^{-1}$)**	**Plasma VTG ($nmol\bullet \mu L^{-1}$)**	**Average fecundity ($eggs\bullet female^{-1}\bullet day^{-1}$)**	**Spawns per Female**	**Eggs per Spawn**	**Reference**
methylT_36	28.53	0.183	19.8	2.0	128.5	[4]
methylT_37	16.87	0.108				
methylT_38	13.31	0.085				
methylT_39	18.44	0.118				
methylT_42	23.67	0.152	11.8	1.0	152.8	
methylT_43	46.95	0.301				
methylT_44	34.3	0.220				
methylT_45	12.52	0.080				
methylT_48	25.21	0.162	26.1	1.3	271.2	
methylT_49	30.28	0.194				
methylT_50	20.84	0.134				
methylT_51	25.45	0.163				
methylT_54	15.96	0.102	2.9	0.5	76.0	
methylT_55	47.49	0.304				
methylT_56	34.36	0.220				
methylT_57	31.53	0.202				
ktc_10532	12	0.077	7.5	2.5	62.7	[5]
ktc_10533	8.5	0.054				
ktc_10534	25.8	0.165				
ktc_10535	19	0.122				
ktc_10562	9.25	0.059	23.9	4.3	118	
ktc_10563	9.68	0.062				
ktc_10564	8.61	0.055				
ktc_10565	20.8	0.133				
ktc_10503	9.21	0.059	10.7	2	112.7	
ktc_10504	7.72	0.049				
ktc_10505	8.91	0.057				
ktc_10592	16.5	0.106	15.7	3.0	109.6	
ktc_10593	12.5	0.080				
ktc_10594	20	0.128				

References cited:

1. Ankley GT, Jensen KM, Makynen EA, Kahl MD, Korte JJ, et al. (2003) Effects of the androgenic growth promoter 17β-trenbolone on fecundity and reproductive endocrinology of the fathead minnow. Environmental Toxicology and Chemistry 22: 1350-1360.
2. Ankley GT, Kahl MD, Jensen KM, Hornung MW, Korte JJ, et al. (2002) Evaluation of the aromatase inhibitor fadrozole in a short-term reproduction assay with the fathead minnow (*Pimephales promelas*). Toxicological Sciences 67: 121-130.
3. Ankley GT, Defoe DL, Kahl MD, Jensen KM, Makynen EA, et al. (2004) Evaluation of the model anti-androgen flutamide for assessing the mechanistic basis of responses to an androgen in the fathead minnow (*Pimephales promelas*). Environmental Science and Technology 38: 6322-6327.
4. Ankley GT, Jensen KM, Kahl MD, Korte JJ, Makynen EA (2001) Description and evaluation of a short-term reproduction test with the fathead minnow (*Pimephales promelas*). Environmental Toxicology and Chemistry 20: 1276-1290.
5. Ankley GT, Jensen KM, Kahl MD, Makynen EA, Blake LS, et al. (2007) Ketoconazole in the fathead minnow (*Pimephales promelas*): reproductive toxicity and biological compensation. Environmental Toxicology and Chemistry 26: 1214-1223.
